# Deciphering intersystem crossing and energy transfer mechanisms in a nonacoordinated ternary europium(iii) complex: a combined spectroscopic and theoretical study†

**DOI:** 10.1039/d4ra06727d

**Published:** 2024-10-15

**Authors:** Houda Al-Sharji, Rashid Ilmi, Willyan F. Oliveira, Balqees S. Al-Saadi, José D. L. Dutra, Osama K. Abou-Zied, Paul R. Raithby, Muhammad S. Khan

**Affiliations:** a Department of Chemistry, Sultan Qaboos University P. O. Box 36, Al Khod 123 Oman rashidilmi@gmail.com abouzied@squ.edu.om msk@squ.edu.om; b Pople Computational Chemistry Laboratory, Department of Chemistry, UFS 49100-000 São Cristóvão Sergipe Brazil diogobios@academico.ufs.br; c Department of Chemistry, University of Bath Claverton Down Bath BA2 7AY UK p.r.raithby@bath.ac.uk

## Abstract

A monochromatic red emitting nonacoordinate organoeuropium complex with the formula [Eu(hfaa)_3_(Ph-TerPyr)] (Eu-1) incorporating hexafluoroacetylacetone (hfaa) primary ligands and a tridentate 4′-phenyl-2,2′:6′,2′′-terpyridine (Ph-TerPyr) ancillary ligand has been synthesized. The complex was characterized by analytical and spectroscopic methods, and its structure was established by single crystal X-ray diffraction (SC-XRD) analysis at low temperature, which explicitly confirms that the coordination sphere is composed of a EuO_6_N_3_ core. Under the UV excitation, Eu-1 displayed typical red emission in solution with a long-excited state lifetime (*τ*_obs_ = 1048.06 ± 9.39 μs) with a good photoluminescence quantum yield (*Q*^L^_Eu_ = 41.14%). We have utilized pump-probe ultrafast transient absorption spectroscopy in tandem with the time-dependent density functional theory (TD-DFT) and the Lanthanide Luminescence Software Package (LUMPAC) to explore the intricate photophysical event that occurs in the vicinity of the ligands of Eu-1 sensitized photoluminescence (PL).

## Abstract 2

Utilizing pump-probe ultrafast transient absorption spectroscopy in tandem with TD-DFT and LUMPAC, we shed light on the intricate photophysical events occurring in the vicinity of the ligands in a [Eu(hfaa)_3_(Ph-TerPyr)] complex.

## Graphical abstract

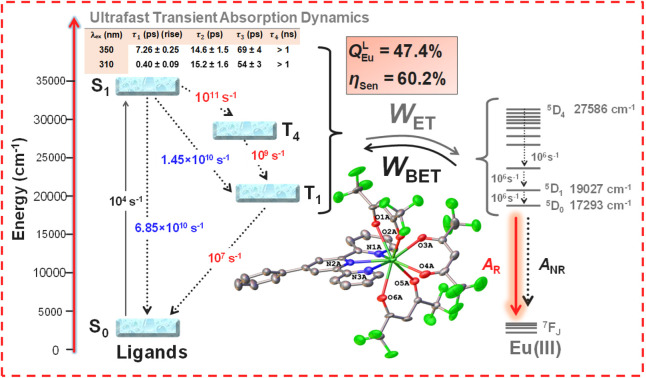

## 1. Introduction

Research on the design and development of stable luminescent organo-lanthanide complexes (OLnCs), particularly complexes of trivalent europium [Eu(iii)], has received wide attention.^1^ This interest stems from their exceptional and unique optical properties, such as intrinsic optically pure red emission,^2^ long excited state lifetimes,^3^ and the potential to reach high quantum yields (up to 90%).^4^ These attributes are difficult to achieve with transition metal complexes, even with thermally activated delayed fluorescent materials.^5^ Thus, OLnCs have been utilized in developing a wide range of innovative technological applications ranging from barcoding to biological assays and imaging.^6^ The emission of Ln(iii) ions primarily results from the Laporte-forbidden intra-configurational 4f–4f electronic transitions,^7^ which display low absorption coefficient (*ε*)/oscillator strength (O.S.) (*ε* ≤ 1–10 M^−1^ cm^−1^/O.S. ≈ 10^−6^).^8^ Consequently, direct excitation of the f-electrons to the emissive state of the Ln(iii) ions is not an efficient strategy. Instead, indirect excitation through the “antenna effect” is more effective.^9^ This involves coordinating Ln(iii) ions with suitable organic ligand(s) that absorb the irradiated light and then efficiently transfer the absorbed energy to the emissive levels of the Ln(iii) ions, causing them to luminesce.^9,10^

In the present study, we report the synthesis, characterization, and photophysical properties of the nonacoordinate ternary Eu(iii)-β-diketonate complex [Eu(hfaa)_3_(Ph-TerPyr)] (Eu-1). The fluorinated primary antenna ligand hfaa featuring six C–F bonds was deliberately chosen to reduce non-radiative quenching of the Eu(iii) excited state, thereby enhancing the complex's PL properties.^2,11^ The tridentate Ph-TerPyr ligand, with its large π-conjugated rigid planar structure and strong chelating ability,^12^ was utilized as the ancillary ligand. Terpyridine and its derivatives are established excellent neutral ligands for Ln(iii) ions, particularly for Eu(iii)/Tb(iii). They form thermodynamically stable complexes and simultaneously sensitize the PL of Ln(iii) ions, leading to improved overall photophysical properties.^6*a*,12*a*^ Despite extensive studies dealing with the synthesis and optical properties of OEuCs comprising β-diketones and heterocyclic ligands (N^N/O^O/N^N^N/O^O^O *etc.*), a detailed study to underpin the intricate ultrafast optical phenomenon occurring in the close vicinity of the ligands responsible for the sensitized PL of Eu(iii) is rare.^13^ Motivated by this and as part of our long-standing research interest in improving the understanding of optical properties of OEuCs, we directed our research efforts to elucidate and pinpoint the energy transfer (ET) mechanism by employing femtosecond ultrafast transient absorption (TA) spectroscopy and theoretical modelling *i.e.*, time-dependent density functional theory (TD-DFT) and LUMPAC.

## 2. Experimental section

### 2.1. Chemical reagents and synthesis of [Eu(hfaa)_3_(Ph-TerPyr)] (Eu-1)

**Fig. 1:**
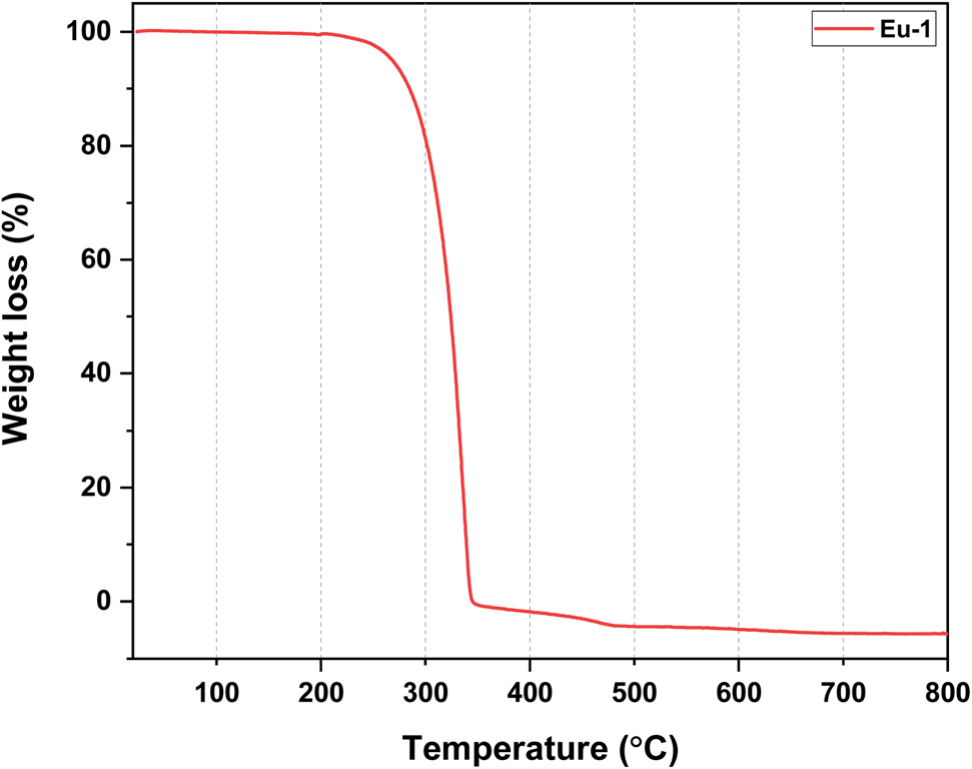
TGA profile of Eu-1 between 25 and 800 °C under N_2_ atmosphere.

All organic and inorganic chemicals and reagents were used as received from commercial sources without further purification. Solvents were dried and distilled before use. Details of the general instrumentation are provided in Section S1 of the ESI†. The synthesis and characterization of Ph-TerPy is detailed in the ESI (Fig. S1 and S2, ESI†). Eu-1 was synthesized by a one-pot method at room temperature (RT). To an ethanolic solution of Hhfaa (0.8046 g, 3.867 mmol), an ethanolic solution of 32% ammonia (0.2 mL) was added with constant stirring. The reaction mixture was covered immediately until all the ammonia vapor dissolved and left to stir for another 30 min. After this period, Ph-TerPyr (0.4007 g, 1.295 mmol) dissolved in a mixture of DCM : EtOH (1 : 1) was added followed by an ethanolic solution of EuCl_3_·6H_2_O (0.4729 g, 1.291 mmol). The reaction mixture was left to stir overnight at room temperature, filtered, and the solvents removed under reduced pressure yielding a white solid. The product was washed with distilled water, followed by hexane, and dried in the air to obtain a pure white solid with a 48% yield. Single crystals of the complex suitable for X-ray Diffraction analysis (CCDC No.: 2370746) were grown at room temperature by slow evaporation of concentrated ethanolic solution (Section SI 2, ESI†). Colour: colourless crystals. Microanalysis calculated for C_36_H_18_EuF_18_N_3_O_6_, C, 39.94; H, 1.68; N, 3.88%; found C, 39.73; H, 1.64; N, 3.79%. FT-IR (KBr pellet; cm^−1^, Fig. S3, ESI†): *ν*(ar C–H st) 3044 cm^−1^; *ν*(C=O st) 1655 cm^−1^; *ν*(C=N st) 1611 cm^−1^; *ν*(C=C st) 1504 cm^−1^; *ν*(C–F st, CF_3_) 1254, 1208 cm^−1^; out-of plane asymmetric *ν*(C–F st) 1144 cm^−1^; in-plane *ν*(C–H bend) 1100 cm^−1^. ESI-MS^+^ (*m*/*z*) = 876.00 for [Eu(hfaa)_2_(Ph-TerPyr)]^+^ (Fig. S4, ESI†). Decomposition temperature (*T*_d_) with 5% weight loss = 298 °C (Fig. 1).

### 2.2. Spectroscopic measurements and photophysical studies

All the spectroscopic measurements of Ph-TerPyr ligand and Eu-1, including optical absorption, excitation, emission spectra, decay profiles, and relative PLQY values, were performed at room temperature in dichloromethane as the solvent medium. Electronic absorption spectra were recorded on a Varian Cary 5000 spectrophotometer whereas, excitation, emission spectra, and decay profiles were carried out utilizing an Edinburgh FS5 fluorometer. A range of important photophysical parameters for Eu-1 such as the *J*–*O* parameters (*Ω*_2_ and *Ω*_4_), radiative (*A*_rad_), nonradiative (*A*_nrad_) decay rates, natural radiative lifetime (*τ*_*r*_), and intrinsic quantum yield (*Q*^Eu^_Eu_) were calculated using the emission spectrum and the *τ*_obs_ value by applying the equations detailed in Section SI 3, ESI.† The ultrafast transient absorption measurements were performed using a femtosecond laser setup as previously described^14^ and is also included in the Section SI 4, ESI.† The details of the theoretical approaches are presented in Section SI 5–SI 7, ESI.†

## 3. Results and discussion

### 3.1. Synthesis, characterization and thermal studies

The nonacoordinated Eu-1 was synthesized by a reported one-pot reaction method.^6*a*^Eu-1 was isolated by reacting Hhfaa, ammonium hydroxide (32% ammonia), Ph-TerPyr, and EuCl_3_·6H_2_O in 3 : 3:1 : 1 molar ratios, respectively, in ethanol at RT. Eu-1 was characterized by elemental analysis, FT-IR spectroscopy, mass spectrometry (MS), and thermogravimetric analysis (TGA), while its solid-state structure was established by single-crystal X-ray analysis (SC-XRD). The elemental analysis result is consistent with the proposed formulation of Eu-1. The ESI-MS spectrum of Eu-1 in DCM (Fig. S4, ESI†) in the positive mode showed an ion peak at *m*/*z* = 876.00 corresponding to Eu-1 after the loss of one hfaa from the coordination sphere as [Eu(hfaa)_2_(Ph-TerPyr)]^+^. The FT-IR spectra of Ph-TerPyr and Eu-1 are displayed in Fig. S1 and S3, ESI.† The spectrum of Eu-1 confirms the presence of coordinated Ph-TerPyr and hfaa ligands. It exhibited a strong absorption peak at 1655 cm^−1^ due to the C=O stretching vibration. C=N and C=C stretching bands of Eu-1 appeared at 1611 cm^−1^ and 1504 cm^−1^, respectively, which are at higher wavenumbers than those of the free Ph-TerPyr ligand (C=N: 1583 cm^−1^ and C=C: 1465 cm^−1^), thus indicating the coordination of the ligand to the Eu(iii) centre. The absorptions at 1254 cm^−1^, 1208 cm^−1^, and 1144 cm^−1^ are related to C–F stretching and out-off plane asymmetric vibrations of the –CF_3_ groups. The thermal stability of Eu-1 was evaluated, and the resultant thermogram is shown in Fig. 1. A close analysis of the thermogram reveals the absence of any considerable weight loss up to 195 °C, reflecting the anhydrous nature of Eu-1. As shown in Fig. 1, Eu-1 exhibited a one-step weight loss with *T*_d_ of 298 °C, implying that Eu-1 has high thermal stability suitable to be employed in the fabrication of electroluminescent devices.

### 3.2. Low-temperature SC-XRD studies

**Fig. 2:**
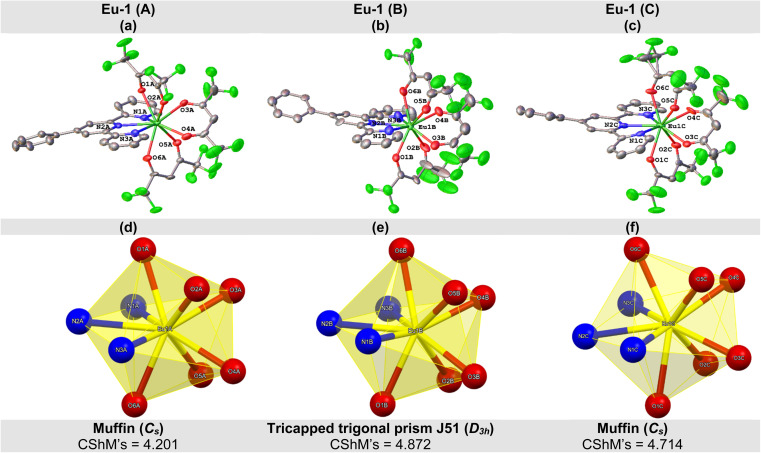
Molecular structure of (a) Eu-1 (A), (b) Eu-1 (B), and (c) Eu-1 (C) with displacement ellipsoids drawn at the 50% probability. Coordination polyhedron of (d) Eu-1 (A), (e) Eu-1 (B), and (f) Eu-1 (C).

The crystal structure of Eu-1 was determined from single-crystal X-ray method at 100 K. The key crystallographic parameters are presented in Table S2, ESI.† An analysis revealed that Eu-1 is a mononuclear complex and crystallizes in the orthorhombic space group *Pca*2_1_ with three independent molecules (Fig. S5, ESI†), Eu-1 (A), Eu-1 (B), and Eu-1 (C), in the asymmetric unit of the unit cell (Fig. 2). In each independent molecule, the central Eu(iii) ion is bonded to six oxygen (O)-atoms of three hfaa ligands and three pyridyl nitrogen (N)-atoms of Ph-TerPyr ancillary ligand forming a N_3_O_6_ coordination sphere. Interestingly, a detailed analysis of three independent molecules further revealed that they are not identical as evidenced from some of the selected bond lengths (Å) and angles (°) given in Tables 1 and S3, ESI† respectively. The three independent molecules of Eu-1 exhibited average Eu–N. and Eu–O bond distances, similar to those reported in analogous complexes.^15^ The three hfaa ligands exhibited asymmetric coordination to the Eu(iii) centres in the three independent molecules, in which the asymmetry in the Eu–O bond distances was found to be 0.079 Å (Eu-1 (A)), 0.089 Å (Eu-1 (B)), and 0.070 Å (Eu-1 (C)). This asymmetric behaviour of β-diketone ligand coordination is consistent with the previously reported hfaa complexes.^11*c*^ The O–Eu–O angles are in the range of 68.8(4) to 73.1(4)° (Eu-1 (A)), 68.8(4) to 77.9(5)° (Eu-1 (B)), and 68.4(4) to 73.1(5)° (Eu-1 (C)). On the other hand, the N–Eu–N (N1–Eu–N2, N2–Eu–N3, and N1–Eu–N3) angles span the range 62.8(4) to 127.0(4)° (Eu-1 (A)), 63.6(5) to 127.3(5)° (Eu-1 (B)), and 63.3(4) to 127.6(4)° (Eu-1 (C)). An analysis of the coordination geometry of the three independent molecules using the SHAPE 2.1 software^16^ showed that Eu-1 (A) and Eu-1 (C) adopted a muffin geometry (*C*_s_; with a deviation of 4.201 [Eu-1 (A)] and 4.714 [Eu-1 (A)] from the idealized muffin geometry, Fig. 2d, f, Table S4, ESI†) while Eu-1 (B) displayed a tricapped trigonal prismatic geometry (*D*_3h_; with a deviation of 4.872 from the idealized tricapped trigonal prism geometry, Fig. 2e and Table S4, ESI†). Moreover, deviation from the respective idealized geometries could be due to the loss of planarity of the Ph-TerPyr ligand as determined by the dihedral angles (DHAs) between the plane of the central pyridyl ring (N2-containing ring) and the planes of the other peripheral pyridyl rings (N1- and N3-containing rings) (Table S5, ESI†).

**Table 1:** Selected bond lengths (Å) of Eu-1 (A), Eu-1 (B), and Eu-1 (C)

Bond	Length (Å)		
	Eu-1 (A)	Eu-1 (B)	Eu-1 (C)
Eu-O(1)	2.420(10)	2.370(12)	2.423(10)
Eu-O(2)	2.452(11)	2.459(12)	2.493(13)
Eu-O(3)	2.370(12)	2.373(14)	2.405(12)
Eu-O(4)	2.449(12)	2.359(13)	2.398(12)
Eu-O(5)	2.472(10)	2.486(13)	2.434(12)
Eu-O(6)	2.401(12)	2.412(14)	2.411(11)
Eu-N(1)	2.574(13)	2.564(14)	2.563(13)
Eu-N(2)	2.577(13)	2.587(14)	2.531(14)
Eu-N(3)	2.580(12)	2.557(16)	2.573(13)
Eu-O_avg_	2.427(44)	2.409(96)	2.427(45)
Eu-N_avg_	2.577(13)	2.569(48)	2.555(8)

A closer inspection of the crystal structure of Eu-1 further revealed the existence of extensive intra- and intermolecular hydrogen (H)-bonding interactions (Fig. S7(a) and Table S6, ESI†). Two types of intramolecular H-bonds were observed in each independent molecule of Eu-1. Firstly, two C–H⋯F interactions between the α-hydrogen atoms (H(24), H(29), and H(34)) of each hfaa ligand with two different fluorine (F)-atoms from the adjacent –CF_3_ groups. However, the H(34) atom in the Eu-1 (A) molecule exhibits only one intra-molecular H-bonding interaction with one F-atom from one adjacent –CF_3_ group. The second type is the C–H⋯O H-bonds, in which the Eu-1 (B) and Eu-1 (C) independent molecules exhibited three C–H⋯O H-bonds between the oxygen atoms (O(2), O(4), and O(5)) of hfaa ligands and the H-atoms (H(15) and H(1)) of the N3- and N1-containing pyridyl rings of the Ph-TerPyr ligand. On the other hand, the Eu-1 (A) molecule only exhibits C–H⋯O H-bonds through its oxygen (O)-atoms (O(2) and O(5)) with the H-atoms (H(15) and H(1)) of the N3- and N1-containing pyridyl rings of the Ph-TerPyr ligand. Regarding the intermolecular H-bonding interactions, several C–H⋯F H-bonds were observed between the F-atoms of the –CF_3_ groups in one Eu-1 molecule and the Ph-TerPyr hydrogen atoms in adjacent Eu-1 molecule, thus forming a 3D network of molecules. The crystal structure analysis of Eu-1 further indicates that the crystallographically independent molecules are linked by intermolecular π–π stacking interactions (Fig. S7(b), ESI†), imparting further stabilization to the structure. The N1-containing pyridyl ring of the Eu-1 (B) molecule (Cg1) exhibits π–π stacking contact with the phenyl ring of adjacent Eu-1 (C) molecule (Cg3) with a separation of 4.109 Å between the centroids of the two rings. On the other hand, the phenyl ring of the Eu-1 (B) molecule (Cg2) is nearly parallel to the N2-containing pyridyl ring of the Eu-1 (C) molecule (Cg4) in which their mean planes intersect with an angle of 16.55° and a centroid-to-centroid distance of 3.878 Å.

During the course of our structural studies, an independent structure determination of Eu-1 has appeared.^17^ In that report, the X-ray data was recorded at room temperature, and the complex crystallised in the monoclinic space group *Pn*, again with three independent molecules in the asymmetric unit. The coordination geometry of each independent Eu centre was described as a spherical capped square antiprism. Since the two unit cells cannot be readily interconverted geometrically, and because of the difference in the assigned space groups, we believe that the crystal in our determination and that in the independent study are polymorphs of each other. Since the computational and spectroscopic studies were carried out on the crystalline material produced in our laboratory, we are relating our findings to the *Pca*2_1_ polymorph.

The ground state geometry of Eu-1 was also determined theoretically by different methods to reproduce the experimental structure. The suitability of the best methods was determined by root mean square deviation (RMSD) values (Table S7, ESI†). Analysis of the RMSD values indicates that the PBE1PBE/TZVP/MWB52 DFT level of theory provided the best structural description (muffin-type (CShMs = 0.986; *C*_s_ point group). The spherical coordinates of the atoms coordinated to the Eu(iii) ion, listed in Table S8† and Eu–O and Eu–N bond lengths are in line with the experimental values.

### 3.3. Analysis and discussion of experimental and theoretical photophysical properties

**Fig. 3:**
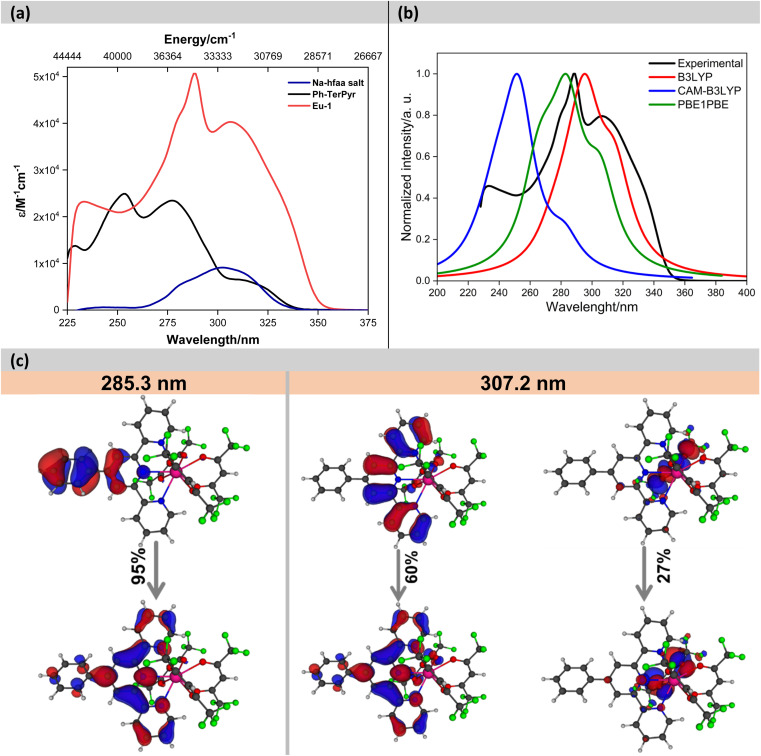
(a) Experimental absorption spectra of the Na-hfaa salt, Ph-TerPyr and Eu-1 in DCM (2 × 10^−5^ M), (b) theoretical absorption spectra, (c) pictures of the natural transition orbitals (NTO) for the main absorption bands of Eu-1 calculated using PBE1PBE/TZVP/MWB52 (DCM). The contribution of each transition is indicated.

The electronic absorption spectra of the Na-hfaa salt, Ph-TerPyr and Eu-1 are displayed in Fig. 3. The Na-hfaa salt showed a broad absorption band in the UV region with *λ*^max^_abs_ ∼302 nm (8873 M^−1^ cm^−1^), while Ph-TerPyr exhibited three main absorption peaks with maxima at 228 (13 694 M^−1^ cm^−1^), 253 (24 902 M^−1^ cm^−1^), 277 (23 450 M^−1^ cm^−1^) nm, and a weaker shoulder at 311 (6515 M^−1^ cm^−1^) nm. These absorption bands could be assigned to the singlet π–π* intraligand (^1^IL) transition of the TerPyr unit and to the π–π* intraligand charge transfer (^1^ICT) transition between the phenyl and TerPyr unit. On the other hand, the absorption spectrum of the Eu-1 displayed two main intense absorption bands peaking at *λ*^max^_abs_ = 288 nm (50 622 M^−1^ cm^−1^) and 306 nm (40 283 M^−1^ cm^−1^), which are formed mainly due to the overlap of the spin allowed π–π* transitions of both ligands (hfaa and Ph-TerPyr). A detailed theoretical study was performed to elucidate the role of ligands in the electronic transitions. An analysis of the results (Fig. 3b and c) suggests that the band shifted to higher wavelengths is dominated by electronic transitions involving molecular orbitals (MOs) primarily centred on the Ph-TerPyr ligand while the most intense absorption band involves electronic transitions encompassing MOs centred on both the hfaa and the neutral Ph-TerPyr ligands.

**Fig. 4:**
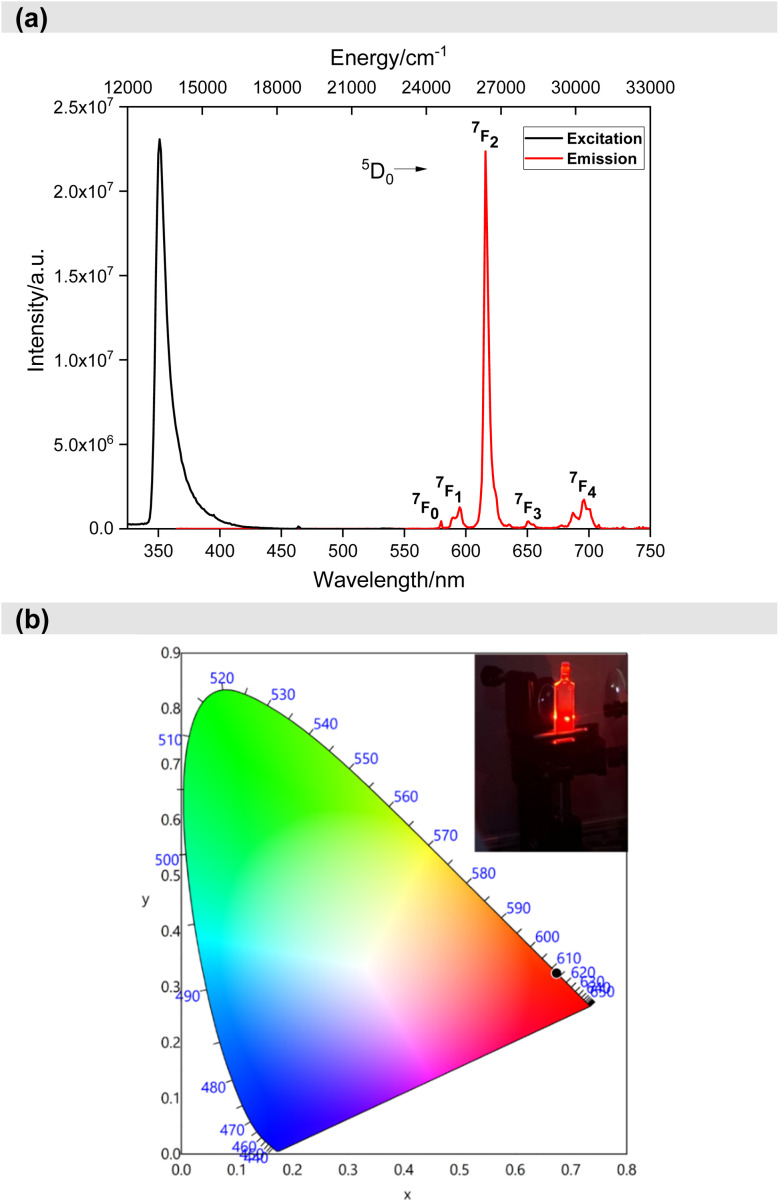
(a) Excitation and emission spectra of Eu-1 in DCM solution (1 × 10^−3^ M) at RT and (b) CIE-1931 chromaticity diagram of Eu-1 with the inset displaying emission photograph of Eu-1 taken during the measurement of ultrafast dynamics.

After understanding the ligand's role in the complex's light absorption properties, the PL properties of Eu-1 were investigated at room temperature, including excitation, emission, excited state lifetime, and PLQY. The excitation spectrum in DCM solution (Fig. 4a) displayed a broad band between 330–425 nm with *λ*^max^_ex_ = 351 nm, attributed to the excitation of the organic chromophores with very faint intraconfigurational ^7^F_0_ → ^5^L_6_ (394 nm) and ^7^F_0_ → ^5^D_2_ (464 nm) transitions implying indirect excitation *via* the well-known antenna mechanism. The emission spectrum (Fig. 4a) was obtained by exciting Eu-1 at *λ*^max^_ex_ = 351 nm and showed typical, well-resolved Eu(iii) five transitions (^5^D_0_ → ^7^F_J_; *J* = 0–4). The photophysical data obtained are summarized in Table 2. Among the five emission transitions, the most intense band in the spectrum was the hypersensitive ED ^5^D_0_ → ^7^F_2_ transition (*λ*^max^_em_ = 616 nm ≈ 16 191.63 cm^−1^), contributing 77.54% to the total integral intensity, resulting in red emission (Colour Purity (CP) = 100%; CIE_*x*,*y*_ = 0.671, 0.325, Fig. 4b) with a FWHM = 4.20 nm, suggesting that Eu-1 could be employed as a red-emitting component in electroluminescent devices.

**Table 2:** Photophysical parameters of Eu-1 in DCM solution (1 × 10^−3^ M) at RT

Photophysical parameters	Experimental	Theoretical	
^5^D_0_ → ^7^F_0_	17 252.11 cm^−1^(0.45%)^a^		
^5^D_0_ → ^7^F_1_	16 860.06 cm^−1^ (6.02%)^a^		
^5^D_0_ → ^7^F_2_	16 191.63 cm^−1^ (77.54%)^a^		
^5^D_0_ → ^7^F_3_	15 330.19 cm^−1^ (2.05%)^a^		
^5^D_0_ → ^7^F_4_	14 407.66 cm^−1^ (13.92%)^a^		
FWHM of ^5^D_0_ → ^7^F_2_	4.20 nm		
Intensity ratio^b^ (*R*_21_)	12.87		
CIE colour coordinates	*x* = 0.674; *y* = 0.325		
Colour Purity^c^ (CP) (%)	100		
*τ*_obs_ (μs)	1048.06 ± 9.39		
*Ω*_2_ d (×10^−20^ cm^2^)	23.09	23.09	
*Ω*_4_ d (×10^−20^ cm^2^)	9.17	9.16	
*A*_rad_ e (s^−1^)	752.41	751.53	
*A*_Nrad_ e (s^−1^)	201.78	202.58	
*τ*_R_ f (μs)	1314	1330	
*Q* ^Eu^_Eu_ g (%)	78.85	78.76	
*Q* ^L^_Eu_ h (%)	41.14	47.4	
Sensitization efficiency^i^ (*η*_sen_) (%)	52.55	60.2	

a Total % contribution of emission intensity relative to ^5^D_0_ → ^7^F_1_ magnetic dipole transition.

b Intensity ratio of electric dipole to magnetic dipole transitions.

c CP of the emitted red colour is determined by 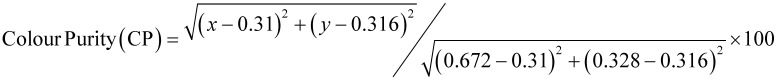.

d *Ω*_2_ and *Ω*_4_ were calculated by the eqn (S1) and (S2).

e *A*_rad_ and *A*_Nrad_were calculated by the eqn (S2)–(S4).

f *τ*_R_ is calculated by eqon (S5).

g *Q* ^Eu^_Eu_ is calculated by the eqn (S6).

h *Q* ^L^_Eu_ is calculated by the eqn (S8).

i *η*_sen_ is calculated by the eqn (S7).

Eu-1 showed a long excited state lifetime (*τ*_obs_) value of 1048.06 ± 9.39 μs in DCM solution (Fig. S5, ESI†) which is more than 3-times higher than [Eu(hfaa)_3_(H_2_O)_2_] (357 ± 5.00 μs), in line with Eu(iii) ternary heteroleptic nonacoordinate complexes but shorter than homoleptic nonacoordinated Eu(iii) complexes (2.94 ms).^18^ This means that the forbidden 4f–4f electronic transitions become more allowed when the symmetry of the coordination sphere around Ln(iii) is reduced and is directly related to the mixing of 4f and 5d orbitals. This observation is well supported by our own work^12*a*^ and that of Bunzli *et al.*^18^ and Hasegawa *et al.*^19^ and is in line with the predictions made from group-theoretical considerations.^20^ Other important photophysical properties (*Ω*_2_ and *Ω*_4_, *A*_rad_, *A*_Nrad_, *τ*_r_, *Q*^Eu^_Eu_, *Q*^L^_Eu_and *η*_sen_) of the sensitized Eu(iii) PL are summarized in Table 2, calculated using eqn (S1)–(S8).† The complex exhibited large *Ω*_2_ and *Ω*_4_ values of 23.09 × 10^−20^ cm^2^ and 9.17 × 10^−20^ cm^2^, respectively (Table 2). The large *Ω*_2_ value of Eu-1 indicates an asymmetric coordination sphere with a highly polarizable chemical environment around the Eu(iii) centre, consistent with the high asymmetric ratio (*R*_21_ = 13.04, Table 2) and the *C*_s_ point group. Moreover, as expected, Eu-1 exhibited a significantly high *Ω*_4_ (9.17 × 10^−20^ cm^2^) value due to extensive long-range effects (hydrogen bonding and π–π stacking). The complex in dichloromethane solution showed large *Q*^L^_Eu_ and *Q*^Eu^_Eu_ values of 41.14% and 78.85%, respectively, resulting in a *η*_sen_ of 52.55%.

### 3.4. Analysis of ultrafast transient absorption dynamics and energy transfer mechanism

**Fig. 5:**
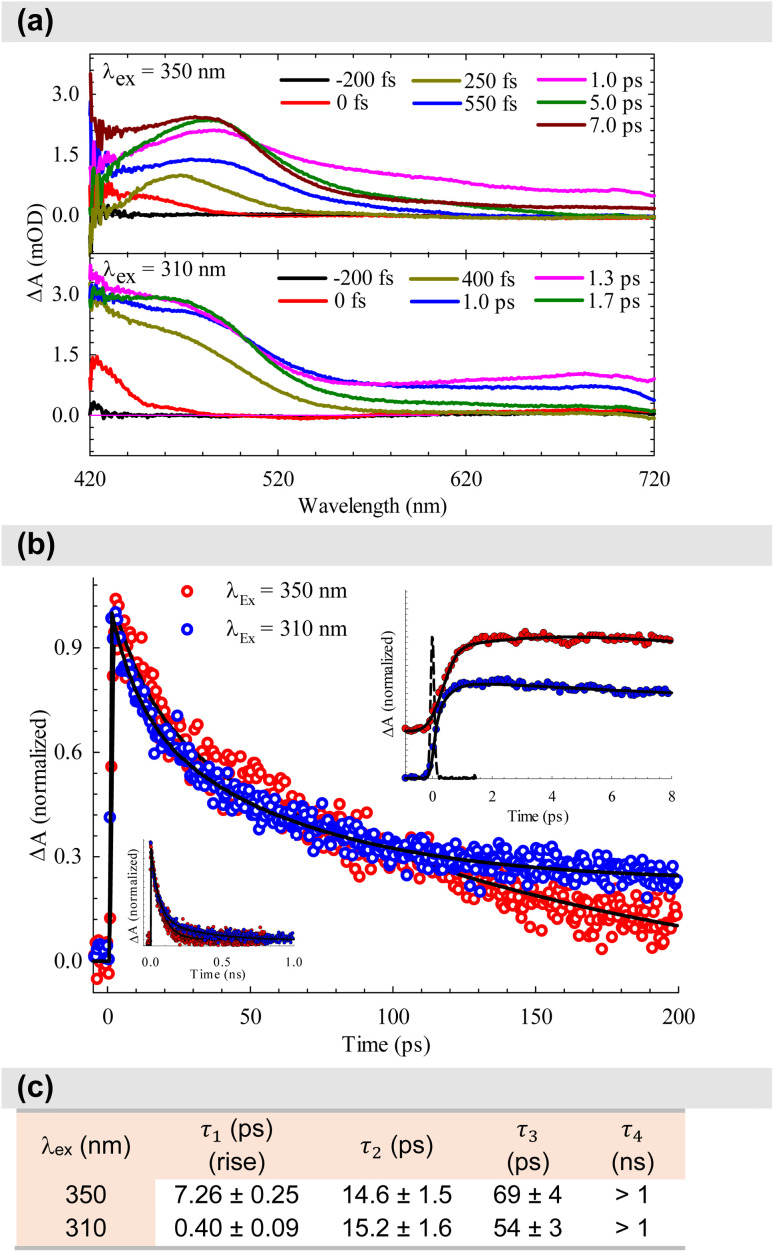
(a) Transient absorption spectra of the complex dissolved in DCM for two pump energies. (b) Dynamics of the complex, derived from the transient absorption spectra shown in (a). The dashed line in the upper inset represents the system response measured from the Raman scattering of the solvent. (c) Time constants derived from the multiexponential fits shown in (b).

After establishing the photophysical properties, we investigated the photophysical events occurring in the excited states. To achieve this, we utilized femtosecond TA spectroscopy to capture the ultrafast dynamics within our system. The data obtained from these experiments were then used to elucidate the most probable ET mechanisms *via* theoretical calculations. Fig. 5a presents snapshots illustrating the spectral changes observed at various time intervals after time zero, following the interaction between the pump (*λ*_ex_ = 350 or 310 nm) and the white-light probe. Our findings reveal an excited state absorption (ESA) band spanning 420–720 nm, demonstrating evolution over time before eventual decay. This band represents absorption from the S_1_ state to higher excited state(s). As time progresses, spectral broadening occurs, indicating the population of numerous vibrational states subsequent to the initial excitation. The spectra in Fig. 5a indicate that excitation at 310 nm leads to greater spectral broadening due to the higher energy states made accessible in both the β-diketone and the PhTerPyr ligands. Further insights into the intricate dynamics of the ESA band can be obtained from the temporal profiles depicted in Fig. 5b. Monitoring the transient dynamics at any spectral region within the ESA peak around 475 nm yielded the same lifetime components. The spectral change with time of this band reflects the nonradiative dynamics in the first excited state since there is no indicative signal of stimulated emission (negative signal). The time constants derived from the multi-exponential fits are outlined in Fig. 5c. Drawing from previous studies on analogous complexes,^13*b*,21^ these four lifetime components are attributed to the following processes: The first component is assigned to internal conversion and vibrational relaxation from higher excited states to S_1_ (τ_1_). This is evident in the build-up of signal intensity (upper inset in Fig. 5b) within this time frame due to more population of the S_1_ state after relaxation from highly excited states. The following two components represent the transient decay of the S_1_ state, with the first decay component (τ_2_) assigned to the nonradiative fate of the excited state to S_0_ and the second decay component (τ_3_) is most likely due to S_1_ → T_1_ intersystem crossing (ISC). The last decay component (τ_4_) is a long lifetime and could not be measured within our detection window. This long component is assigned to the dynamics of the triplet state T_1_.

A substantial reduction in τ_1_ is evident for excitation at 310 nm (from 7.26 to 0.40 ps). This decline in lifetime signifies a more efficient nonradiative internal conversion/vibrational relaxation process at higher energies, facilitated by increased accessibility of a large number of vibrational states. There is no observed effect on τ_2_, within the experimental uncertainty, which is consistent with the nature of this process as a decay of the S_1_ state to S_0_. The excitation energy should not affect this process, since it takes place from the lowest vibrational levels in S_1_. τ_3_ is reduced by ∼20% for 310 nm excitation. This reduction highlights the more efficient mechanism of ISC at higher energy. The last lifetime component τ_4_ can be assigned to T_*n*_ ← T_1_ absorption. The contribution from this component to the overall transient is <5% at 350 nm excitation, while this ratio increases to about 20% at 310 nm excitation (transients are shown in the lower inset in Fig. 5b). This observation is consistent with the more efficient ISC at higher energies, resulting in more population of the T_1_ state.

To elucidate the energy transfer mechanism and calculate the ligand–metal ET rates, we first determined the values of energy and *R*_L_ (Table S9, ESI†). We applied these in LUMPAC using the Malta's model. These rates incorporate contributions from both the Ex and direct CI mechanisms. The latter contribution was calculated using the FED intensity parameters (*Ω*^FED^_λ_) provided by the QDC model^22^ (Table S10, ESI†). The larger value of the dynamic coupling (DC) intensity parameters (*Ω*^DC^_λ_) suggests that the emission of the metal centre is influenced by the polarizabilities and symmetry of the surrounding chemical environment. Experimentally, ultrafast TA spectroscopy revealed that the S_1_ → S_0_ decay rate (6.85 × 10^10^ s^−1^) is faster than the S_1_ → T_1_ decay rate (1.45 × 10^10^ s^−1^). Considering only these two channels for depopulating S_1_, it follows that non-radiative decay to S_0_ dominates the depopulation of S_1_. Consequently, T_1_ is not efficiently populated, and even if the T_1_ → ^5^D_0,1_ transfer rates are significant, the quantum yield will remain limited. Moreover, the T_1_ → S_0_ decay rate does not significantly impact the T_1_ population due to the limited population of T_1_. For instance, tests have shown that, in this context, the theoretical quantum yield reaches a maximum value of approximately 17.5%, while the experimental value is 41.14%, resulting in a relative error of 57.5% between the theoretical and experimental values. Therefore, considering the level system commonly addressed in the literature, based only on the S_0_, S_1_, and T_1_ states, it is not possible to fully explain the theoretical quantum yield of the complex.

These observations led to two hypotheses: (i) the energy transfer rate from S_1_ state to an excited state of Eu(iii) is greater than the S_1_ → S_0_ decay rate, or (ii) S_1_ undergoes a fast additional decay to a given T_*n*_ state, from which T_1_ is populated more efficiently than through S_1_ → T_1_. The first hypothesis appears unlikely, as calculations revealed that the highest ET rate involving S_1_ is of the order of 10^6^ s^−1^ (Table 3). This rate is associated with the ^7^F_1_ → ^5^G_2_ transition that is governed by the Ex. mechanism. The other significant electronic transition rates from S_1_ involve the ^7^F_1_ → ^5^D_3_ and ^7^F_1_ → ^5^G_3_ transitions, primarily mediated by the CI mechanism. Additionally, Table 3 shows that the highest ET rates are due to the T_1_ → ^5^D_0_ and T_1_ → ^5^D_1_ acceptor channels, with rates of the order 10^8^ s^−1^. This supports the role of T_1_ in the electronic excitation of the lanthanide ion. These channels correspond to the ^7^F_0_ → ^5^D_1_ and ^7^F_1_ → ^5^D_0_ excitations in Eu(iii) and are governed by the Ex mechanism. Therefore, the second hypothesis is more plausible. Moreover, characterization of the ligands' excited states further reveals that T_1_, T_2_, and T_3_ are nearly degenerate (with a difference of approximately 300 cm^−1^). The analysis of the MOs involved in the electronic transitions of these triplet states shows that they differ only in the redistribution of electron density among the three coordinated hfaa ligands and the Ph-TerPyr ligand. Furthermore, analysis of Table S9 and Fig. S9† indicate that the triplet state with significant contributions from the auxiliary ligand is T_4_. The proposed ET scheme for the Eu-1 complex can be simplified as follows: S_0_ → S_1_ → T_4_ → T_1_ → Eu(iii). This scheme highlights the crucial role of the neutral ligand in the ET process, beyond its function of preventing solvent molecules from coordinating to the metal centre. However, along with the large pool of complexes where the triplet pathway is dominant, there are also instances of direct singlet energy transfer pathways.^24^ Further details of the intricate sensitization processes and ET mechanisms are available from the excellent work by Bünzli,^25^ Ward^26^ and Malta.^23^

**Table 3:** Energy transfer rates calculated using the PBE1PBE/TZVPPD/MWB52 TD-DFT (DCM) results with Malta's model^23^ implemented in LUMPAC

Donor	Acceptor	*W* ^CI^_ET_ (s^−1^)	*W* ^EX^_ET_ (s^−1^)	*W*_BET_ (s^−1^)
S_1_	^7^F_0_ → ^5^D_0_^a^	1.10 × 10^0^	0.0	1.80 × 10^−32^
	^7^F_0_ → ^5^D_1_	0.0	1.39 × 10^3^	9.25 × 10^−26^
	^7^F_0_ → ^5^L_6_	2.91 × 10^3^	0.0	2.55 × 10^−12^
	^7^F_0_ → ^5^G_6_	2.39 × 10^3^	0.0	1.96 × 10^−9^
	^7^F_0_ → ^5^D_4_	1.15 × 10^5^	0.0	5.17 × 10^−6^
	^7^F_1_ → ^5^D_0_	0.0	1.63 × 10^2^	4.48 × 10^−31^
	^7^F_1_ → ^5^D_1_	8.02 × 10^2^	3.77 × 10^−1^	8.99 × 10^−27^
	^7^F_1_ → ^5^D_2_	0.0	2.19 × 10^3^	3.20 × 10^−21^
	^7^F_1_ → ^5^D_3_	1.45 × 10^5^	0.0	2.04 × 10^−13^
	^7^F_1_ → ^5^L_6_	6.22 × 10^2^	0.0	9.14 × 10^−14^
	^7^F_1_ → ^5^L_7_	2.80 × 10^3^	0.0	5.80 × 10^−11^
	^7^F_1_ → ^5^G_2_	0.0	2.00 × 10^6^	4.92 × 10^−8^
	^7^F_1_ → ^5^G_3_	6.80 × 10^5^	0.0	5.03 × 10^−8^
	^7^F_1_ → ^5^G_6_	9.93 × 10^2^	0.0	1.37 × 10^−10^
	^7^F_1_ → ^5^G_5_	1.03 × 10^4^	0.0	1.49 × 10^−9^
T_1_	^7^F_0_ → ^5^D_0_^a^	4.89 × 10^0^	0.0	2.51 × 10^−10^
	^7^F_0_ → ^5^D_1_	0.0	2.07 × 10^8^	4.32 × 10^1^
	^7^F_0_ → ^5^L_6_	3.34 × 10^−1^	0.0	9.16 × 10^5^
	^7^F_0_ → ^5^G_6_	3.96 × 10^−2^	0.0	1.02 × 10^8^
	^7^F_0_ → ^5^D_4_	5.57 × 10^−1^	0.0	7.83 × 10^10^
	^7^F_1_ → ^5^D_0_	0.0	4.22 × 10^8^	3.63 × 10^−3^
	^7^F_1_ → ^5^D_1_	5.63 × 10^2^	9.29 × 10^4^	3.29 × 10^−3^
	^7^F_1_ → ^5^D_2_	0.0	1.94 × 10^7^	8.89 × 10^4^
	^7^F_1_ → ^5^D_3_	7.53 × 10^1^	0.0	3.31 × 10^5^
	^7^F_1_ → ^5^L_6_	1.18 × 10^−1^	0.0	5.44 × 10^4^
	^7^F_1_ → ^5^L_7_	1.31 × 10^−1^	0.0	8.53 × 10^6^
	^7^F_1_ → ^5^G_2_	0.0	2.30 × 10^7^	1.77 × 10^15^
	^7^F_1_ → ^5^G_3_	1.64 × 10^1^	0.0	3.81 × 10^9^
	^7^F_1_ → ^5^G_6_	2.72 × 10^−2^	0.0	1.18 × 10^7^
	^7^F_1_ → ^5^G_5_	2.58 × 10^−1^	0.0	1.17 × 10^8^
T_4_	^7^F_0_ → ^5^D_0_^a^	4.99 × 10^−2^	0.0	1.69 × 10^−20^
	^7^F_0_ → ^5^D_1_	0.0	3.51 × 10^6^	4.85 × 10^−9^
	^7^F_0_ → ^5^L_6_	1.09 × 10^−1^	0.0	1.97 × 10^−3^
	^7^F_0_ → ^5^G_6_	2.69 × 10^−2^	0.0	4.58 × 10^−1^
	^7^F_0_ → ^5^D_4_	6.86 × 10^−1^	0.0	6.37 × 10^2^
	^7^F_1_ → ^5^D_0_	0.0	2.42 × 10^6^	1.37 × 10^−13^
	^7^F_1_ → ^5^D_1_	1.16 × 10^1^	1.30 × 10^3^	3.05 × 10^−13^
	^7^F_1_ → ^5^D_2_	0.0	9.63 × 10^5^	2.92 × 10^−5^
	^7^F_1_ → ^5^D_3_	2.39 × 10^1^	0.0	6.95 × 10^−4^
	^7^F_1_ → ^5^L_6_	3.17 × 10^−2^	0.0	9.68 × 10^−5^
	^7^F_1_ → ^5^L_7_	6.01 × 10^−2^	0.0	2.58 × 10^−2^
	^7^F_1_ → ^5^G_2_	0.0	1.43 × 10^7^	7.29 × 10^6^
	^7^F_1_ → ^5^G_3_	1.66 × 10^1^	0.0	2.54 × 10^1^
	^7^F_1_ → ^5^G_6_	1.53 × 10^−2^	0.0	4.38 × 10^−2^
	^7^F_1_ → ^5^G_5_	1.64 × 10^−1^	0.0	4.93 × 10^−1^

a The ^7^F_0_ → ^5^D_0_ transition was included in the calculations by means of a *J*-mixing of 5% involving the ^7^F_0_ and ^7^F_2_ states.

**Fig. 6:**
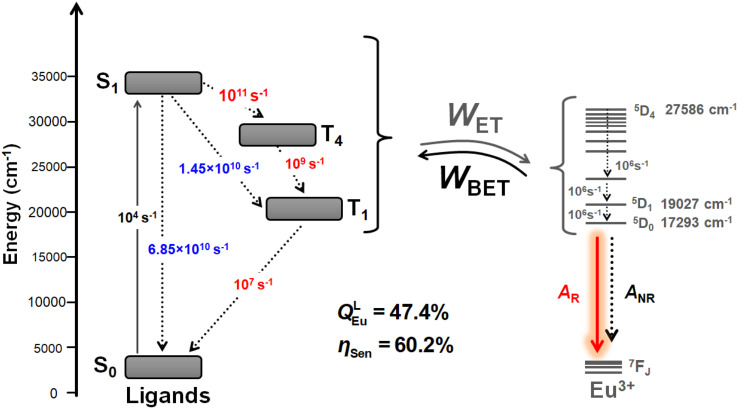
Representative energy level diagram highlighting the levels and channels included in the modelling of energy transfer in Eu-1.

Fig. 6 presents a schematic energy level diagram for Eu-1, highlighting the contributions of S_0_, S_1_, T_4_ and, T_1_ of the ligands to the ET process leading to sensitized Eu(iii) luminescence. To quantify the energetic population of all states involved in the ET modelling, the experimental decay rates for the S_1_ → S_0_ and S_1_ → T_1_ transitions were used (values highlighted in blue). The unknown rate constants for the S_1_ → T_4_, T_4_ → T_1_, and T_1_ → S_0_ transitions were subsequently adjusted to accurately reproduce the experimentally determined sensitization efficiency (values highlighted in red). This procedure is based on a methodology previously employed in other studies by our research group.^6*a*,12*a*,27^Fig. 6 shows that rates of 10^11^ s^−1^, 10^9^ s^−1^, and 10^7^ s^−1^ for the S_1_ → T_4_, T_4_ → T_1_, and T_1_ → S_0_ transitions, respectively, provide a theoretical emission quantum yield of 47.4% and a sensitization efficiency of 60.2%. More precise adjustments of these rates could yield values closer to the experimental results. Nevertheless, the proposed energy level diagram, along with the adjusted and experimental rates, offer detailed insights into the importance of the ligands for the ET process of Eu-1.

## 4 Conclusion

In summary, a red-emitting nonacoordinated Eu-1 was successfully synthesized and characterized. The structure of Eu-1 was determined by the SC-XRD. Analysis of the crystal structure further revealed the presence of three independent molecules in the asymmetric unit within the unit cell, out of which two adopted the muffin coordination geometry while the third displayed a tricapped trigonal prismatic geometry. Under excitation, Eu-1 exhibited typical red emission which showed large *Q*^L^_Eu_ and *Q*^Eu^_Eu_ that resulted in a sensitization efficiency of 52.55%. A thorough analysis of femtosecond TA experiment results revealed that the S_1_ → S_0_ decay rate (6.85 × 10^10^ s^−1^) is faster than the intersystem crossing *i.e.*, S_1_ → T_1_ decay rate (1.45 × 10^10^ s^−1^) that led to assumptions that S_1_ undergoes an additional fast decay to a given T_*n*_ state, from which T_1_ is populated more efficiently than through S_1_ → T_1_ with the following path S_0_ → S_1_ → T_4_ → T_1_ → Eu(iii) of the sensitized Eu(iii). This is further supported by analysis of the MOs^20^ which showed that the T_1_–T_3_ are almost degenerate (with a difference of approximately 300 cm^−1^) and only differ in the redistribution of electron density among the primary and ancillary ligand with significant contribution from T_4_ of Ph-TerPyr. This scheme highlights the crucial role of the neutral ligand in the ET process, beyond its function of preventing solvent molecules from coordinating to the metal centre.

## Data availability

The data supporting this article have been included as part of the ESI.†

## Conflicts of interest

The authors have no conflicts of interest to declare.

## Supplementary Material

RA-014-D4RA06727D-s001

RA-014-D4RA06727D-s002
