# Supplementary material for: Deciphering intersystem crossing and energy transfer mechanisms in a nonacoordinated ternary europium(iii) complex: a combined spectroscopic and theoretical study
Source: RSC Adv. 2024 Oct 15;14(44):32573–82. doi: 10.1039/d4ra06727d (PMC11474895; doi:10.1039/d4ra06727d)
Supplement: RA-014-D4RA06727D-s001 [file RA-014-D4RA06727D-s001.pdf]

Supplementary Information (SI) for RSC Advances.
This journal is © The Royal Society of Chemistry 2024

Deciphering Intersystem Crossing and Energy Transfer Mechanisms in A Nonacoordinated Ternary Europium(III) Complex: A Combined Spectroscopic and Theoretical Study

Houda Al-Sharji,[a] Rashid Ilmi,*[a] Willyan F. Oliveira,[b] Balqees S. Al-Saadi,[a] José D. L. Dutra,*[b] Osama K Abou-Zied*[a], Paul R. Raithby*[c] and Muhammad S Khan*[a]

[a]Department of Chemistry, Sultan Qaboos University, P. O. Box 36, Al Khod 123, Oman

[b]Pople Computational Chemistry Laboratory, Department of Chemistry, UFS, 49100-000 São Cristóvão, Sergipe, Brazil

[c]Department of Chemistry, University of Bath, Claverton Down, Bath, BA2 7AY, UK

E-mail addresses and ORCID ID of corresponding authors:

Rashid Ilmi (RI)	: rashidilmi@gmail.com; 0000-0002-5165-5977
Osama K Abou-Zied (OKA)	: abouzied@squ.edu.om; 0000-0003-0497-8412
Muhammad S. Khan (MSK)	: msk@squ.edu.om; 0000-0001-5606-6832
José D. L. Dutra (JDLD)	: diogobios@academico.ufs.br; 0000-0003-2123-2209
Paul R. Raithby	: p.r.raithby@bath.ac.uk; 0000-0002-2944-0662

Electronic Supporting Information

SI 1. Experimental Section: General instrumentation

The melting point of the **Ph-TerPyr** ligand was measured using the Stuart Scientific melting point SMP 10 apparatus. The Fourier transform infrared (FT-IR) spectra of pure solid samples were recorded on PerkinElmer Frontier FT-IR spectrometer in the range 400-4000 cm^{-1}; the solid samples were compressed as KBr disks using a Specac manual hydraulic press. Elemental analysis was performed on Euro EA-CHN Elemental Analyser in the Department of Chemistry, Sultan Qaboos University. ^1H and ^{13}C NMR spectra of Ph-TerPyr ligand were recorded on Bruker Biospin Avance III HD (700 MHz) FT-NMR spectrometer with $SiMe_4$ as the internal reference at the Central Analytical and Applied Research Unit (CAARU) at Sultan Qaboos University. Mass spectra were obtained using an LCMS-8040, Shimadzu-Japan coupled to a triple quadruple tandem mass spectrometer equipped with electrospray ionization (ESI). Thermal stability of the complexes was determined by thermogravimetric Analysis (TGA) and differential thermogravimetric analysis (DTA) using SDT Q600 (V20.9 Build 20) thermogravimetric analyzer and differential scanning calorimeter heated in the temperature range between 50-800 ℃ at a heating rate of 20 ℃/min under dinitrogen (N_2) atmosphere.

SI 2. Single-crystal X-ray diffraction (SC-XRD)

A suitable crystal of **Eu-1** was selected and mounted on a MiTeGen microloop in fomblin oil on a Bruker APEX-II CCD diffractometer. The crystal was kept at 100 K during data collection. Using Olex2[1], the structure was solved with the ShelXT[2] structure solution program using Intrinsic Phasing and refined with the XL[3] refinement package using Least Squares minimization. The crystal data, data collection and refinement parameters are presented in Tables S1 – S8, in the Supporting Information. Crystallographic data have been deposited with the Cambridge Crystallographic Data Centre as supplementary publication number CCDC 2370746. Copies of the data can be obtained free of charge via https://www.ccdc.cam.ac.uk/conts/retrieving.html, or from the Cambridge Crystallographic Data Centre, 12 Union Road, Cambridge CB2 1EZ, UK. Fax: +44 1223 336033: or e-mail: deposit@ccdc.cam.ac.uk.

SI 3. Equations applied to Calculate Photophysical Parameters

$$\Omega_\lambda^{\exp} = \frac{3\hbar A_{Rad}\left[{}^5D_0 \rightarrow {}^7F_J\right]}{32e^2\pi^3\chi v\left[{}^5D_0 \rightarrow {}^7F_J\right]^3 \left|\left\langle {}^5D_0 \left\| U^{(\lambda)} \right\| {}^7F_J \right\rangle\right|^2} \quad \text{Eq. S1}$$

$$A_{Rad} = \sum_{J=0}^{4} A_{Rad}\left[{}^5D_0 \rightarrow {}^7F_J\right] \quad \text{Eq. S2}$$

$$A_{Rad}\left[{}^5D_0 \rightarrow {}^7F_J\right] = \frac{v\left[{}^5D_0 \rightarrow {}^7F_1\right]}{v\left[{}^5D_0 \rightarrow {}^7F_J\right]} \times \frac{A\left[{}^5D_0 \rightarrow {}^7F_J\right]}{A\left[{}^5D_0 \rightarrow {}^7F_1\right]} A_{Rad}\left[{}^5D_0 \rightarrow {}^7F_1\right] \quad \text{Eq. S3}$$

$$A_{tot} = \frac{1}{\tau_{obs}} = A_{Rad} + A_{NRad} \quad \text{Eq. S4}$$

$$\tau_R = 1/A_{Rad}$$ Eq. S5

$$Q_{Eu}^{Eu} = \frac{\tau_{obs}}{\tau_{Rad}} = \frac{A_{Rad}}{A_{Rad} + A_{NRad}}$$ Eq. S6

$$\eta_{sen} = \frac{Q_{Eu}^{L}}{Q_{Eu}^{Eu}}$$ Eq. S7

The relative quantum yield of **Eu-1** (Q_{Eu}^{L}) was determined relative to [Eu(btfa)$_3$(H$_2$O)$_2$] (Q_r = 1.9%) where btfa is the anion of 4,4,4-trifluoro-1-phenyl-1,3-butanedione. Q_s was calculated using the following Eq S8.

$$Q_{Eu}^{L} = \frac{A_r \times I_s \times \eta_s^2}{A_s \times I_r \times \eta_r^2} \times Q_r$$ Eq. S8

$A, I,$ and η represent absorbance at the excitation wavelength, integrated intensity of the emission spectra, and refractive index, respectively. DCM has η value of 1.424.

SI 4. Pump-probe Ultrafast Transient Absorption Spectroscopy

The pump and probe pulses were obtained using a regenerative amplified Ti:Sapphire laser (Libra, Coherent). The Libra generates compressed laser pulses (70 fs pulse width) with an output power of 4.26 W at a repetition rate of 5 kHz and centered at 800 nm. The output beam was split into two parts. The major portion of the output pulse was used to pump a Coherent OPerA Solo (Light Conversion Ltd.) optical parametric amplifier to generate spectrally tunable light spanning the range 240−2600 nm and is used as the pump beam. The remaining small portion of the laser output was focused on a sapphire crystal to generate a white light continuum in the range 420-800 nm, which is used as the probe beam in a Helios transient absorption spectrometer (Ultrafast Systems, LLC). The probe light was measured by a fiber optic that is coupled to a multichannel spectrometer with a CMOS sensor in the range 350-850 nm. Chirp in the white light continuum probe was minimized by using parabolic mirrors. Rotational contribution to the overall excited state decay kinetics was removed by depolarizing the pump beam using a depolarizer (DPU-25, Thorlabs). The pump pulse was attenuated to ~100 nJ to avoid multiphoton excitation. The pump and probe pulses were focused on the sample and the temporal delay of the probe pulse was varied using a computer-controlled optical delay stage. Kinetic traces at appropriate wavelengths were assembled from the time-resolved spectral data. Surface Xplorer software (supplied by Ultrafast Systems) was used for data analysis. In order to adjust the zero delay for each wavelength and to get the chirp-corrected spectrum, we carried out the transformation process using the software program (Surface Xplore). The instrument response function (IRF) was measured from Raman scattering to be ~ 120 fs. The samples in solution for the lifetime measurements were prepared

in 2 mm fused silica cuvettes (Spectrocell Inc.) and were stirred during the experiment. All measurements were conducted at 22.0 ± 0.5 °C.

SI 5. Optimization of the Ground State Geometry and Singlet ($^1\pi\pi^*$) and Triplet ($^3\pi\pi^*$) State Energy Levels of the Complexes

Since the studied system exhibits crystallographic structure, this structure was employed to assess whether the DFT approach at the PBE1PBE/TZVP/MWB52 theory level, already applied in previous works published by us,[4] presents higher accuracy when compared to other methods. The Root-Mean-Square Deviation (RMSD) was used as metric, considering both all atoms in the structure and only those within the coordination polyhedron as references. Accordingly, the crystallographic structure was employed as the starting structure in all geometry optimization calculations, and the SVP and TZVPPD basis sets were also evaluated. SVP treats the electronic structure of the hydrogen atom using (4s1p)/[2s1p] and applies (7s4p1d)/[3s2p1d] for the atoms of the second period of the p-block. For TZVP, the (5s1p)/[3s1p] and (11s6p1d)/[5s3p1d] basis sets are used, and TZVPPD considers the (5s3p1d)/[3s3p1d] and (12s7p3d1f)/[6s3p3d1f] bases, respectively. The objective of applying these three basis sets was to evaluate the impact of increasing components in the basis functions on the structural modeling of the studied complex. For representing the electronic structure of the europium atom, the MWB(52)[5] effective core potential (ECP) was used, where 52 electrons are in the core, and the 11 remaining electrons are described by (7s6p5d)/[5s4p3d]. The calculations were performed using the ORCA program, version 5.0.3.[6] In addition to applying the DFT approach, the GFN-2 model[7] implemented in the xTB program,[8] together with the RM1 semiempirical model[9] using the MOPAC program 22.0.6,[10] were also applied. In all calculations, the charge and spin multiplicity of the complex were set to zero and singlet, respectively.

The PBE1PBE/TZVP/MWB52 geometry was used to calculate the ligand-centered singlet and triplet excited states, as it was the most consistent with the experimentally determined structure. To assess the performance of various density functionals in predicting absorption spectra, TD-DFT calculations were employed using a diverse set of over 50 density functionals implemented in the ORCA program.[6] The TZVP basis set in conjunction with the MWB52 ECP were applied, and the implicit solvent effect was also considered in the calculations. The energies of the singlet states, along with their corresponding oscillator strengths, were used to obtain the theoretical absorption spectrum through a Lorentzian fit with an arbitrary half-height bandwidth of 15 nm. The calculated absorption spectra were then compared with the experimental one. Among the various density functionals tested, the PBE1PBE/TZVP/MWB52 level of theory provided the TD-DFT result that exhibited the greatest agreement with the experimental absorption spectrum of the **Eu-1** complex.

SI 6. Modelling of the Energy transfer (ET) Mechanism

The TD-DFT results obtained via PBE1PBE/TZVP/MWB52 method were utilized to calculate ligand-metal energy transfer (ET) rates according to Malta's model by following the protocol [11] reported by our research group,[4] and implemented in the LUMPAC software package.[12] The model is based on Fermi's golden rule[13] and Hamiltonian operator that include contributions from the Coulomb interaction (CI, Eq. S9) and exchange (Ex, Eq. S11) mechanisms.

$$W_{ET}^{CI} = \frac{2\pi}{\hbar}\frac{e^2 S_L F}{G(2J+1)}\sum_{\lambda=2,4,6}\Lambda_\lambda \left\langle \psi' J' \left\| U^{(\lambda)} \right\| \psi J \right\rangle^2 \qquad \text{Eq. S9}$$

$$\Lambda_\lambda = 2\Omega_\lambda^{FED}\left(1-\sigma_1\right)^2\left(\frac{1}{R_L^6}\right) + \left\langle r^\lambda \right\rangle^2 \left\langle 3 \left\| C^{(\lambda)} \right\| 3 \right\rangle^2 \left(1-\sigma_\lambda\right)^2 \left(\frac{\lambda+1}{\left(R_L^{\lambda+2}\right)^2}\right) \qquad \text{Eq. S10}$$

$$W_{ET}^{Ex} = \frac{8\pi}{3\hbar}\frac{e^2}{R_L^4}\frac{\left(1-\sigma_0\right)^2 F}{G(2J+1)}\left\langle \psi' J' \left\| S \right\| \psi J \right\rangle^2 \sum_m \left\langle \Psi_{N-1}\Pi \left| \sum_j r_j C_0^{(1)}(j) s_{-m}(j) \right| \Psi_{N-1}\Pi^* \right\rangle^2 \qquad \text{Eq. S11}$$

$$F = \frac{1}{\hbar\gamma_L}\sqrt{\frac{ln 2}{\pi}}\, e^{-\left(\frac{\Delta}{\hbar\gamma_L}\right)^2 ln 2} \qquad \text{Eq. S12}$$

$$R_L = \frac{\sum_i c_i^2 R_{L_i}}{\sum_i c_i^2} \qquad \text{Eq. S13}$$

$$\left(1-\sigma_0\right) \approx 0.05\left(\frac{R_{\min}}{R_L}\right)^{7/2} \qquad \text{Eq. S14}$$

The TD-DFT results are useful for quantifying the *F* term, given by Eq. S12, and the distance between the energy donor and acceptor centers involved in the ligand-metal ET channel (R_L, Eq. S13). The Δ term present in Eq. S12 is the energy difference between the donor and acceptor excited states involved in the ET process. This energy difference was calculated using the TD-DFT singlet and triplet excited states' energies of the ligands and the excited states energies of Eu(III) taken from the work of Carnall and coworkers.[14] The excited states of the Eu(III) ion considered were $^7F_0$, $^7F_1$, $^5D_0$, $^5D_1$, $^5D_2$, $^5D_3$, $^5D_4$, $^5L_6$, $^5L_7$, $^5G_2$, $^5G_3$, $^5G_5$, and $^5G_6$. The ligand bandwidth at half-height (γ_L) was assumed a typical value of 3250 cm^{-1}.

The R_L quantity given by Eq. S13 is determined as a function of the atomic orbital coefficients of the ith atom (c_i) contributing to the ligand state and the distance from a given *i* atom to the metal ($R_{L,i}$). In addition, R_L is also used to estimate the shielding factor $\left(1-\sigma_0\right)$ by Eq. S14,

where R_{min} is the smallest distance between the atoms of the coordination polyhedron and the Eu(III) ion.

The $\langle \psi' J' \| U^{(\lambda)} \| \psi J \rangle$ and $\langle \psi' J' \| S \| \psi J \rangle$ matrix elements provide $|\Delta J|$ = 2, 4, 6 and $|\Delta J|$ = 0, 1 (J = J'= 0 excluded) as selection rules for the CI and Ex mechanisms, respectively. To include excitations from the lowest excited states $^7F_0$ and $^7F_1$ to higher excited states of Eu(III), values of thermal populations equal to 0.64 ($^7F_0$) and 0.33 ($^7F_1$) were considered, assuming a temperature of 300 K.[15]

The Ω_λ^{FED} term in Eq. 10 is exclusively due to the contribution of the forced electric dipole (FED) mechanism from the well-established J-O theory. Ω_λ^{FED} is calculated using the (R_j, θ_j, ϕ_j) spherical coordinates of the atoms directly connected to Eu(III). The Ω_λ^{calc} parameters were adjusted to reproduce the experimental values of the J-O intensity parameters of the complex by applying the *QDC* model[16] implemented into LUMPAC.

The backward ET rate from a given state of Eu(III) to a ligand-excited state was calculated by multiplying the respective forward ET rate by the Boltzmann factor, $\exp\left(-|\Delta| / k_B T\right)$, where k_B is the Boltzmann constant, and T is the room temperature. All other quantities appearing in Eq. 9 and Eq. S10 are listed in **Table S1** together with their brief description.

SI 7. Radiative emission rate (A_R) and PLQY

The theoretical photoluminescence quantum yield (PLQY) was calculated in terms of the energetic population of the emitting state of Eu(III) ($\eta_{^5D_0}$) and of the absorber state of the ligand (S_0) using the Eq. S15.

$$Q_{Eu}^{L} = \frac{A_R \eta_{^5D_0}}{\varphi \eta_{S_0}} \qquad \text{Eq. S15}$$

$$A_R = \frac{32 e^2 \pi^3 \chi}{3\text{h}(2J+1)} \sum_{\lambda=2,4,6} \nu[^5D_0 \rightarrow {}^7F_{J=\lambda}]^3 \Omega_\lambda \left| \langle {}^5D_0 \| U^{(\lambda)} \| {}^7F_{J=\lambda} \rangle \right|^2 + \frac{32 \pi^3 n^3 \nu[^5D_0 \rightarrow {}^7F_1]^3}{3\text{h}} S_{md} \qquad \text{Eq. S16}$$

$$\frac{d\eta_i}{dt} = -\sum_{i \neq j} W_{ji} \eta_j + \sum_{i \neq j} W_{ij} \eta_i = 0 \qquad \text{Eq. S17}$$

where A_R is the theoretical radiative emission rate (Eq. S16) and is calculated as a function of the Judd-Ofelt intensity parameters determined by the *QDC* model. The Lorentz local-field correction was estimated with the expression $\chi = n(n^2+2)^2/9$, where n is the refractive index. The ligand-metal ET rates calculated with LUMPAC, and the ligands decay rates determined

using transient ultrafast spectroscopy, were applied to a postulated energy levels system to calculate the energy population of each ith state considered in the ET modeling (η_i). The system of rate equations describing the energy population variation of the involved states was solved under steady-state conditions (Eq. S17). Absorption rate from the fundamental singlet to a given excited singlet of the ligand. A typical value of 10^4 s^{-1} was considered for the absorption rate from the fundamental singlet to a given excited singlet of the ligand (φ).

The quantities in Eq. S15 – S17 used in the calculation of the emission quantum yield and the theoretical radiative emission rate with LUMPAC are shown in **Table SA**.

Table SA: Brief description of some important quantities used in the calculation of emission quantum yield and radiative emission rate with LUMPAC.

Quantity	Description	Value
$\nu[^5D_0 \rightarrow ^7F_J]$	Energy of the barycentre of the $^5D_0 \rightarrow ^7F_2$, $^5D_0 \rightarrow ^7F_4$, and $^5D_0 \rightarrow ^7F_6$ transitions	The values were taken from the work of Carnall and co-workers[14]
n	Refractive index of the medium	To represent the DCM solvent, a value of 1.424 was assumed
S_{md}	Magnetic dipole strength of the $^5D_0 \rightarrow ^7F_1$ transition[17]	9.6×10^{-42} esu^2cm^2
$\left\|\left\langle ^5D_0 \left\| U^{(\lambda)} \right\| ^7F_{J=\lambda} \right\rangle\right\|^2$	Unit operator matrix elements	The values were taken from the work of Carnall and co-workers[14]
W_{ij} and W_{ji}	Energy transfer rate from the i to the j state (W_{ij}) and the backward rate (W_{ji})	The rates were calculated using the models of Malta or typical values were assumed

SI 8. Synthesis of ancillary ligand (Ph-TerPyr)

To a stirred mixture of benzaldehyde (2.73 g, 25.7 mmol) and 2-acetylpyridine (6.23 g, 51.4 mmol) in 200 ml of ethanol (EtOH), sodium hydroxide (NaOH, 2.06 g, 51.4 mmol) and 25% ammonia solution (10 mL) were added. The reaction mixture was stirred for 24 h at 25 ℃. The precipitate was isolated by vacuum filtration and washed with a plenteous amount of cold EtOH. Recrystallization from dichloromethane (CH_2Cl_2, DCM):EtOH (1:1) mixture afforded colourless crystals of Ph-TerPyr (35.5% yield). Microanalysis calculated for $C_{21}H_{15}N_3$, C, 81.53; H, 4.89; N, 13.58%; found C, 81.70; H, 4.69; N, 13.20%. FT-IR (KBr pellet; cm^{-1}, Figure S1): ν(ar C-H st) 3048, 3010 cm^{-1}; ν(C=N st) 1583 cm^{-1}; ν(C=C st, 1465 cm^{-1}. ESI-MS^+ (m/z) = 332.20 for $[M+Na]^+$ (Figure S2). Melting point (T_m) = 207-209 °C.

Table S1: Key quantities for energy transfer rates calculation with the models of Malta implemented in LUMPAC.

Quantity	Description	Value
J	Total angular momentum quantum	Depends on the excited state of the Eu(III) ion considered in the process of ET
G	Degeneracy of the ligand initial state	1 for singlet state ($^1\pi\pi^*$)<br>3 for triplet state ($^3\pi\pi^*$)
$\left\langle 3 \middle\| C^{(\lambda)} \middle\| 3 \right\rangle$	Racah tensor operators	$\left\langle 3 \middle\| C^{(2)} \middle\| 3 \right\rangle$ = -1.366<br>$\left\langle 3 \middle\| C^{(4)} \middle\| 3 \right\rangle$ = 1.128<br>$\left\langle 3 \middle\| C^{(6)} \middle\| 3 \right\rangle$ = -1.270
$\left\langle r^{\lambda} \right\rangle$	Radial integrals for Eu(III)[18]	$\left\langle r^{2} \right\rangle$ = 0.9175<br>$\left\langle r^{4} \right\rangle$ = 2.0200<br>$\left\langle r^{6} \right\rangle$ = 9.0390
$(1-\sigma_{\lambda})$	Shielding field owing to the *5s* and *5p* filled orbitals [19]	σ_2 = 0.600<br>σ_4 = 0.139<br>σ_6 = 0.900
S_L	Ligand dipole strength[19]	10^{-35} (e.s.u.)^2<br>(singlet donor state)<br><br>10^{-40} (e.s.u.)^2<br>(triplet donor state)
$\left\langle \Psi_{N-1}\Pi \middle\| \sum_j r_j C_0^{(1)}(j) s_{-m}(j) \middle\| \Psi_{N-1}\Pi^* \right\rangle^2$	Squared matrix element of the coupled dipole and spin operators [20]	10^{-36} (e.s.u.)^{2}cm^2
$\left\langle \psi' J' \middle\| U^{(\lambda)} \middle\| \psi J \right\rangle$	Unit operator matrix elements	The values taken from the work of Carnall and coworkers [14]
$\left\langle \psi' J' \middle\| S \middle\| \psi J \right\rangle$	Spin operator matrix elements	The values taken from the work of Kasprzycka and coworkers [15]

Table S2: Crystal data and structure refinement for **Eu-1**.

Empirical formula	$C_{36}H_{18}EuF_{18}N_3O_6$
Formula weight	1082.49
Temperature (K)	100
Crystal system	orthorhombic
Space group	$Pca2_1$
a (Å)	17.648(3)
b (Å)	30.273(5)
c (Å)	22.438(4)
α (°)	90
β (°)	90
γ (°)	90
Volume ($Å^3$)	11988(3)
Z	12
ρ_{calc} (g/cm^3)	1.799
μ (mm^{-1})	1.702
F(000)	6336.0
Crystal size (mm^3)	0.55 × 0.31 × 0.175
Radiation	MoKα (λ = 0.71073)
2Θ range for data collection (°)	1.346 to 57.402
Index ranges	$-21 \le h \le 23$, $-35 \le k \le 40$, $-30 \le l \le 30$
Reflections collected	174199
Independent reflections	30923 [R_{int} = 0.0407, R_{sigma} = 0.0303]
Data/restraints/parameters	30923/1907/1755
Goodness-of-fit on F^2	1.079
Final R indexes [I>=2σ (I)]	R_1 = 0.0954, wR_2 = 0.2334
Final R indexes [all data]	R_1 = 0.1048, wR_2 = 0.2444
Largest diff. peak/hole (e $Å^{-3}$)	10.83/-2.82
Flack parameter	0.49(2)

Table S3: Selected bond angles (°) of **Eu-1 (A)**, **Eu-1 (B)**, and **Eu-1 (C)**.

Bond	Eu-1 (A)	Eu-1 (B)	Eu-1 (C)	Bond	Eu-1 (A)	Eu-1 (B)	Eu-1 (C)
	Angle (°)				Angle (°)		
**O(1)-Eu-O(2)**	69.7(4)	68.8(4)	68.4(4)	**O(4)-Eu-O(2)**	71.8(4)	69.6(5)	70.3(4)
**O(1)-Eu-O(3)**	76.5(4)	77.4(4)	72.8(4)	**O(4)-Eu-O(3)**	73.1(4)	77.9(5)	73.1(5)
**O(1)-Eu-O(4)**	135.5(4)	136.6(4)	131.7(4)	**O(4)-Eu-O(5)**	69.5(4)	73.5(5)	71.2(4)
**O(1)-Eu-O(5)**	128.5(4)	126.5(5)	124.1(4)	**O(4)-Eu-O(6)**	82.0(4)	74.9(5)	79.6(5)
**O(1)-Eu-O(6)**	140.4(4)	145.0(4)	147.1(4)	**O(4)-Eu-N(1)**	136.7(4)	144.5(5)	141.5(5)
**O(1)-Eu-N(1)**	68.4(4)	69.8(5)	67.2(4)	**O(4)-Eu-N(2)**	148.3(4)	138.1(5)	145.0(4)
**O(1)-Eu-N(2)**	68.4(4)	70.6(4)	73.3(4)	**O(4)-Eu-N(3)**	90.7(4)	79.8(5)	85.4(5)
**O(1)-Eu-N(3)**	95.6(4)	96.1(5)	101.4(4)	**O(5)-Eu-N(1)**	68.8(4)	71.0(5)	71.2(4)
**O(2)-Eu-O(5)**	134.5(4)	128.6(5)	132.5(4)	**O(5)-Eu-N(2)**	115.1(4)	120.0(4)	118.8(4)
**O(2)-Eu-N(1)**	136.3(4)	137.0(5)	135.1(4)	**O(5)-Eu-N(3)**	133.4(4)	136.8(6)	133.8(4)
**O(2)-Eu-N(2)**	110.4(4)	111.3(5)	108.7(4)	**O(6)-Eu-O(2)**	127.6(4)	130.2(5)	127.6(4)
**O(2)-Eu-N(3)**	69.0(4)	68.6(6)	68.5(4)	**O(6)-Eu-O(5)**	68.8(4)	68.9(5)	69.6(4)
**O(3)-Eu-O(2)**	75.4(4)	71.0(5)	74.4(4)	**O(6)-Eu-N(1)**	93.4(4)	91.2(5)	94.6(4)
**O(3)-Eu-O(5)**	71.3(4)	67.2(5)	68.7(4)	**O(6)-Eu-N(2)**	72.0(4)	74.7(4)	74.1(4)
**O(3)-Eu-O(6)**	138.2(4)	133.3(4)	135.6(4)	**O(6)-Eu-N(3)**	66.8(4)	71.7(6)	67.2(4)
**O(3)-Eu-N(1)**	83.2(4)	89.4(5)	86.1(4)	**N(1)-Eu-N(3)**	127.0(4)	127.3(5)	127.6(4)
**O(3)-Eu-N(2)**	138.7(4)	143.6(5)	141.6(4)	**N(2)-Eu-N(1)**	64.3(4)	63.8(5)	64.5(4)
**O(3)-Eu-N(3)**	144.0(4)	138.5(5)	141.7(4)	**N(2)-Eu-N(3)**	62.8(4)	63.6(5)	63.3(4)

Table S4: A comparative continues shape measures (CShMs) of the crystallographic coordination polyhedron for **Eu-1 (A)**, **Eu-1 (B)**, and **Eu-1 (C)** calculated with the SHAPE software.

Shape	Symmetry	Calculated results		
		Eu-1 (A)	Eu-1 (B)	Eu-1 (C)
Enneagon (EP-9)	D_{9h}	30.909	23.545	29.953
Octagonal pyramid (OPY-9)	C_{8v}	19.237	17.445	19.085
Heptagonal bipyramid (HBPY-9)	D_{7h}	19.421	20.390	19.104
Johnson triangular cupola J3 (JTC-9)	C_{3v}	17.623	13.596	17.454
Capped cube J8 (JCCU-9)	C_{4v}	10.613	12.406	11.304
Spherical-relaxed capped cube (CCU-9)	C_{4v}	11.795	12.951	12.418
Capped square antiprism J10 (JCSAPR-9)	C_{4v}	5.717	6.958	5.923
Spherical capped square antiprism (CSAPR-9)	C_{4v}	5.159	5.984	5.213
Tricapped trigonal prism J51 (JTCTPR-9)	D_{3h}	6.767	**4.872**	7.227
Spherical tricapped trigonal prism (TCTPR-9)	D_{3h}	4.963	6.748	5.396
Tridiminished icosahedron J63 (JTDIC-9)	C_{3v}	15.498	14.785	15.266
Hula-hoop (HH-9)	C_{2v}	11.838	12.964	12.193
Muffin (MFF-9)	C_s	**4.201**	6.365	**4.714**

Table S5: Dihedral angles (°) between the central pyridyl ring plane (B) and the peripheral pyridyl and phenyl rings planes.

Ring Planes	Dihedral angle (°) Eu-1 (A)	Eu-1 (B)	Eu-1 (C)
**B and A**	16.00	14.96	21.22
**B and C**	2.85	10.85	5.84
**B and D**	27.61	24.65	22.40

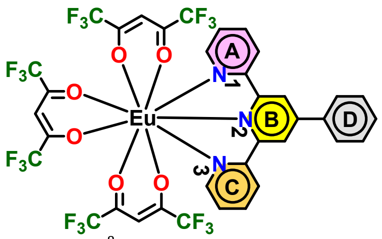

Table S6: Hydrogen bonding interactions in **Eu-1**. d: distances (Å); <: angles (°); D: donor atom and A: acceptor atom.

D-H···A	d(D-H)	d(H···A)	d(D···A)	<(DHA)
C(19A)-H(19A)···F(13C)[#1]	0.951	2.608	3.532	163.76
C(12C)-H(12C)···F(16A)[#2]	0.951	2.613	3.536	163.78
C(21C)-H(21C)···F(16A)[#2]	0.950	2.619	3.537	162.88
C(21A)-H(21A)···F(16C)[#2]	0.949	2.379	3.205	145.26
C(34A)-H(34A)···F(5A)[#2]	0.949	2.418	3.343	164.53
C(17B)-H(17B)···F(3A)[#3]	0.951	2.643	3.492	148.90
C(3A)-H(3A)···F(1B)[#3]	0.949	2.574	3.101	115.31
C(4A)-H(4A)···F(2B)[#3]	0.950	2.626	3.279	126.28
C(3B)-H(3B)···F(4A)[#4]	0.949	2.523	3.320	141.55
C(34C)-H(34C)···F(5C)[#5]	0.951	2.512	3.455	171.16
C(9B)-H(9B)···F(2C)[#6]	0.950	2.614	3.553	169.95
C(12B)-H(12B)···F(2C)[#6]	0.950	2.493	3.356	151.03
C(7C)-H(7C)···F(17B)[#6]	0.950	2.551	3.263	131.86
C(19B)-H(19B)···F(17C)[#7]	0.951	2.663	3.165	113.52
C(24C)-H(24C)···F(3C)[#3]	0.950	2.396	2744	101.29
C(24C)-H(24C)···F(4C)[#3]	0.950	2.650	2.885	94.53
C(29C)-H(29C)···F(8C)[#3]	0.949	2.451	2.776	99.81
C(29C)-H(29C)···F(12C)[#3]	0.949	2.336	2.696	101.83
C(34C)-H(34C)···F(14C)[#3]	0.951	2.518	2.797	96.93
C(34C)-H(34C)···F(17C)[#3]	0.951	2.396	2.717	99.28
C(15C)-H(15C)···O(2C)[#3]	0.949	2.640	2.960	100.21
C(15C)-H(15C)···O(4C)[#3]	0.949	2.626	3.285	126.91
C(1C)-H(1C)···O(5C)[#3]	0.951	2.429	2.981	116.76
C(24A)-H(24A)···F(2A)[#3]	0.950	2.326	2.694	102.36
C(24A)-H(24A)···F(5A)[#3]	0.950	2.521	2.794	96.60
C(29A)-H(29A)···F(8A)[#3]	0.950	2.391	2.751	102.04
C(29A)-H(29A)···F(12A)[#3]	0.950	2.346	2.721	102.97
C(34A)-H(34A)···F(18A)[#3]	0.949	2.310	2.694	103.46
C(15A)-H(15A)···O(2A)[#3]	0.949	2.580	2.973	105.24
C(1A)-H(1A)···O(5A)[#3]	0.951	2.478	2.958	111.23
C(24B)-H(24B)···F(3B)[#3]	0.950	2.387	2.723	100.32
C(24B)-H(24B)···F(4B)[#3]	0.950	2.508	2.791	97.21
C(29B)-H(29B)···F(8B)[#3]	0.950	2.430	2.770	100.78
C(29B)-H(29B)···F(12B)[#3]	0.950	2.401	2.732	100.06
C(34B)-H(34B)···F(14B)[#3]	0.951	2.514	2.791	96.78

C(34B)-H(34B)···F(18B)#3	0.951	2.236	2.601	101.69
C(15B)-H(15B)···O(2B)#3	0.950	2.667	2.943	97.33
C(15B)-H(15B)···O(4B)#3	0.950	2.432	3.099	127.13
C(1B)-H(1B)···O(5B)#3	0.950	2.521	3.006	111.78

Symmetry transformations: (#1) x,y,z; 1-x,-y,-1/2+z (#2) x,y,z; 1.5-x,y,-1/2+z (#3) x,y,z; x,y,z (#4) x,y,z; 1/2+x,1-y,z (#5) 1-x,-y,-1/2+z; -1/2+x,-y,-1+z (#6) 1-x,-y,-1/2+z; 1-x,-y,-1/2+z (#7) 1-x,-y,-1/2+z; -1/2+x,-y,z

Table S7: Root Mean Square Deviation (RMSD) for geometries optimized with different approaches, taking the experimental structure as a reference, calculated both for all atoms in the structure and just the atoms in the coordination polyhedron.

**Method**	**RMSD (Å)**	
	**All atoms**	**Coordination polyhedron**
PBE1PBE/SVP/MWB52	0.5361	0.1051
PBE1PBE/TZVP/MWB52	**0.5372**	**0.0960**
PBE1PBE/TZVP/MWB52 (DCM)	0.5484	0.1106
PBE1PBE/TZVPPD/MWB52	0.6344	0.1002
GFN-2	0.6548	0.1572
RM1	0.5905	0.1373

Table S8: Spherical coordinates of the atoms of the coordination polyhedron of **Eu-1** calculated at the PBE1PBE/TZVP/MWB52 DFT level of theory.

**Atom**	***R* (Å)**	**θ (°)**	**ϕ (°)**
O2 (hfaa 1)	2.4567	17.02	209.95
O3 (hfaa 1)	2.4819	51.12	21.82
O4 (hfaa 2)	2.4073	76.48	102.38
O5 (hfaa 2)	2.4156	119.04	40.24
O6 (hfaa 3)	2.4884	138.95	137.93
O7 (hfaa 3)	2.4545	147.85	277.22
N8 (Ph-TerPyr)	2.5891	84.41	188.42
N9 (Ph-TerPyr)	2.6236	81.42	251.45
N10 (Ph-TerPyr)	2.5887	87.05	314.66

Table S9: Energy, electronic transitions, distance from energy donor to acceptor center (R_L) of the S_1, T_1 and T_4 states predicted using the PBE1PBE/TZVP/MWB52 (DCM) TD-DFT results with the help of LUMPAC.

**State**	**Energy (cm^{-1})**	**R_L (Å)**	**Major Contribution**	**Total**
S_1	32554.6	3.9822	HOMO-2→LUMO (50.31%)<br>HOMO-7→LUMO+2 (11.33%)<br>HOMO-7→LUMO+3 (5.33%)	66.96%
T_1	22233.7	3.7787	HOMO-4→LUMO+1 (36.42%)<br>HOMO-3→LUMO+1 (26.76%)<br>HOMO→LUMO+1 (14.18%)<br>HOMO-4→LUMO+2 (5.57%)	82.93%
T_4	26160.9	4.2959	HOMO-2→LUMO+4 (21.94%)<br>HOMO-2→LUMO+3 (19.55%)<br>HOMO-2→LUMO+2 (13.42%)	60.53%

			HOMO-3→LUMO (5.62%)	

Table S10: Theoretical intensity parameters calculated using the QDC model along with the ZDO electronic density (*q*), electrophilic superdelocalizability (*SE*), charge factor (*g*) and polarizability (α) of the atoms of the coordination polyhedron of **Eu-1**.

**Ligand atom**	***QDC* parameters**			
	Q = 0.0313 au^{-1}: D = 59.4598 au^{-1}·Å^3:C = 21.6393 Å^3 **D/C = 2.75 au^{-1}**			
	***q* (au)**	***SE* (au)**	***g***	**α (Å^3)**
O2 (hfaa 1)	6.3384	-0.2212	0.1987	8.4849
O3 (hfaa 1)	6.3336	-0.2996	0.1985	3.8267
O4 (hfaa 2)	6.3304	-0.2984	0.1984	3.8936
O5 (hfaa 2)	6.3286	-0.2709	0.1983	5.5346
O6 (hfaa 3)	6.3315	-0.3463	0.1984	1.0512
O7 (hfaa 3)	6.3414	-0.3441	0.1987	1.1815
N8 (Ph-TerPyr)	5.2011	-0.3272	0.1630	2.1868
N9 (Ph-TerPyr)	5.1959	-0.3365	0.1628	1.6296
N10 (Ph-TerPyr)	5.2060	-0.2604	0.1632	6.1586
	Ω_2^{FED} = 0.0011×10^{-20} cm^2: Ω_4^{FED} = 0.0076×10^{-20} cm^2 Ω_6^{FED} = 0.0146×10^{-20} cm^2<br><br>Ω_2^{DC} = 23.1340×10^{-20} cm^2: Ω_4^{DC} = 9.6786×10^{-20} cm^2 Ω_6^{DC} = 0.1802×10^{-20} cm^2			

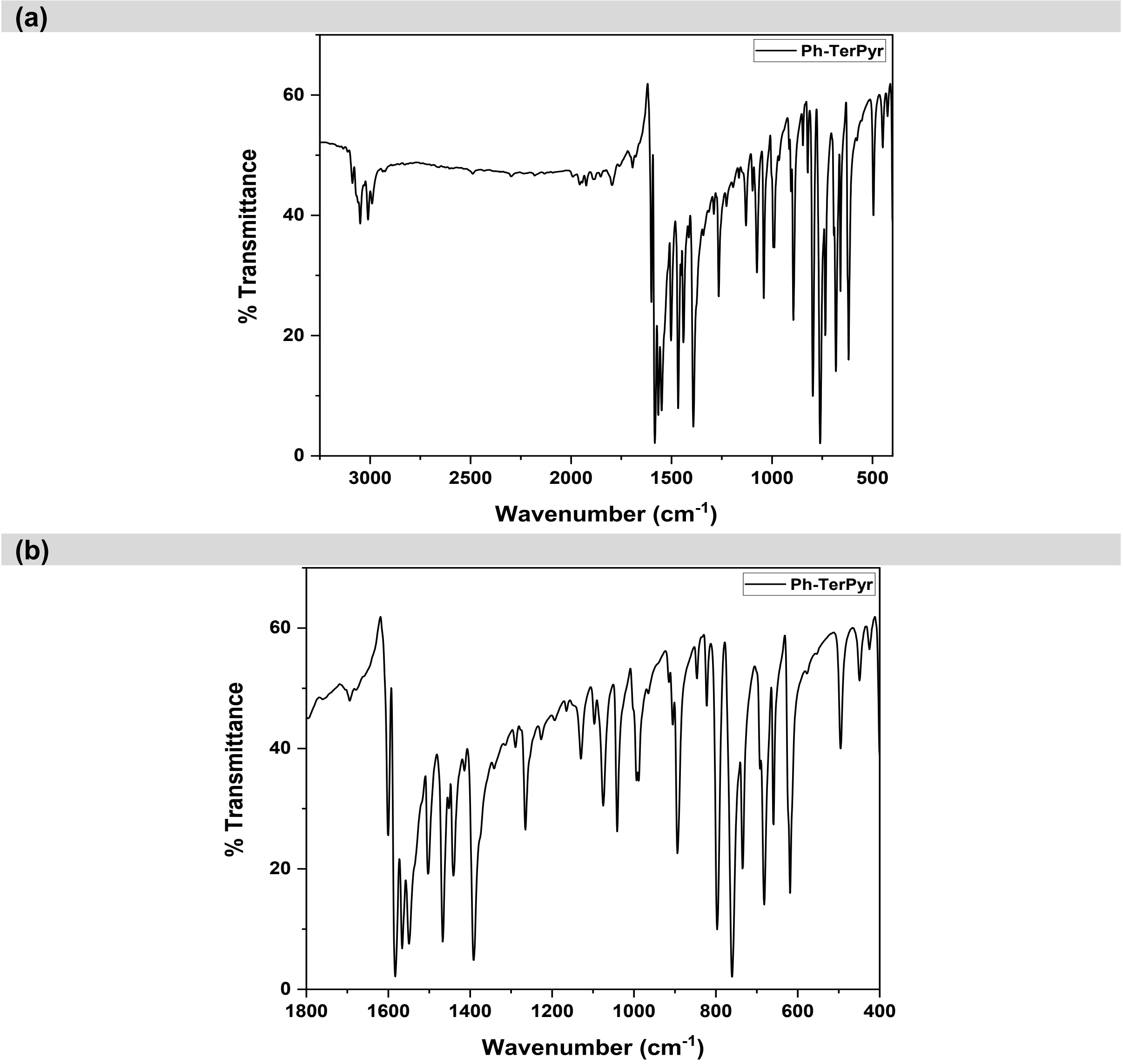

Figure S1: **(a)** FT-IR spectra of the Ph-TerPyr ligand and **(b)** expansion of the region between 1800 – 400 cm^{-1}.

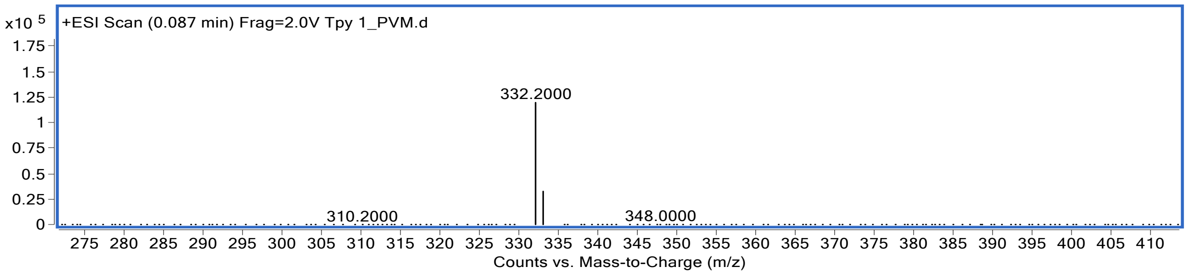

Figure S2: $^{+}$ESI-MS spectrum of the Ph-TerPyr ligand in DCM.

(a)

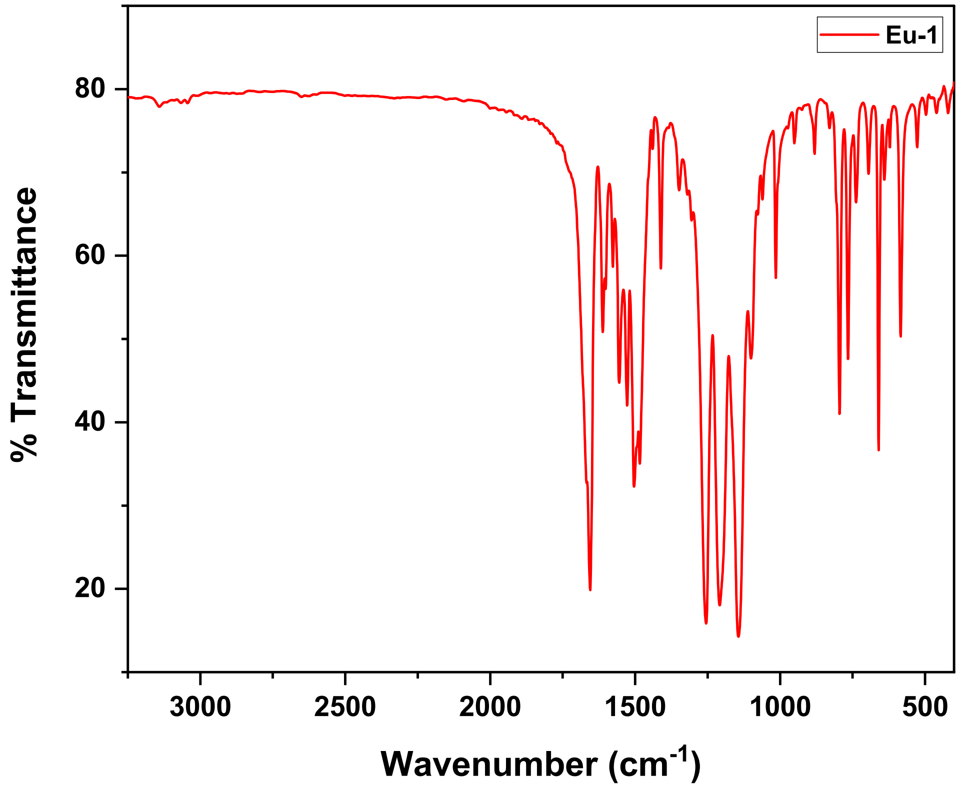

(b)

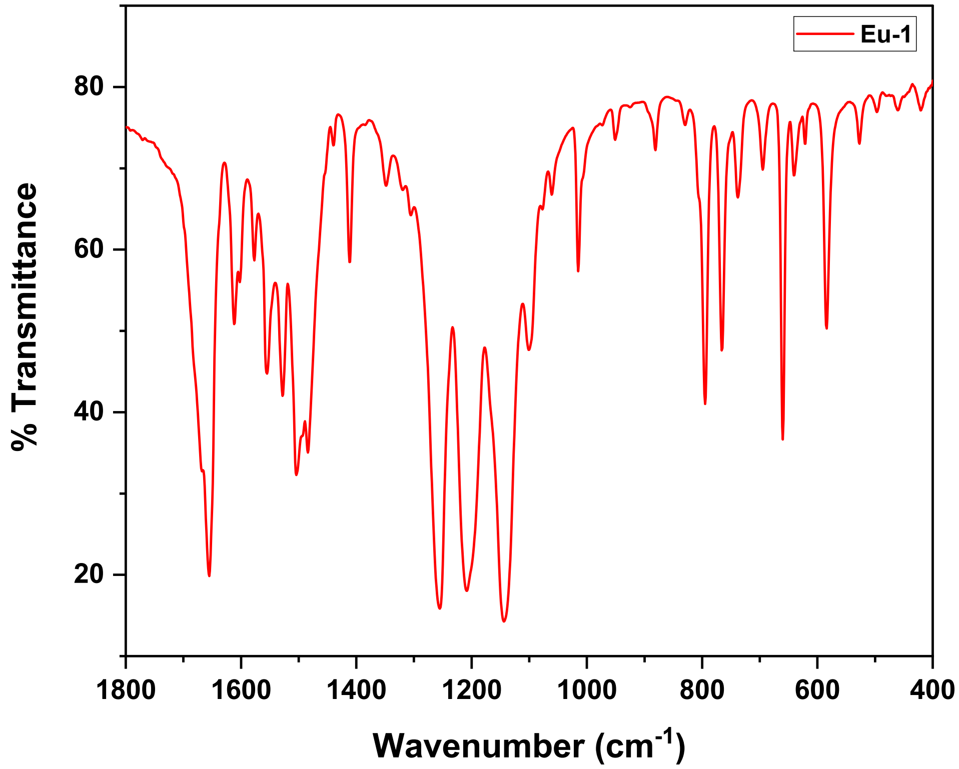

Figure S3: (a) FT-IR spectra of **Eu-1** and **(b)** expansion of the region between 1800 – 400 cm^{-1}.

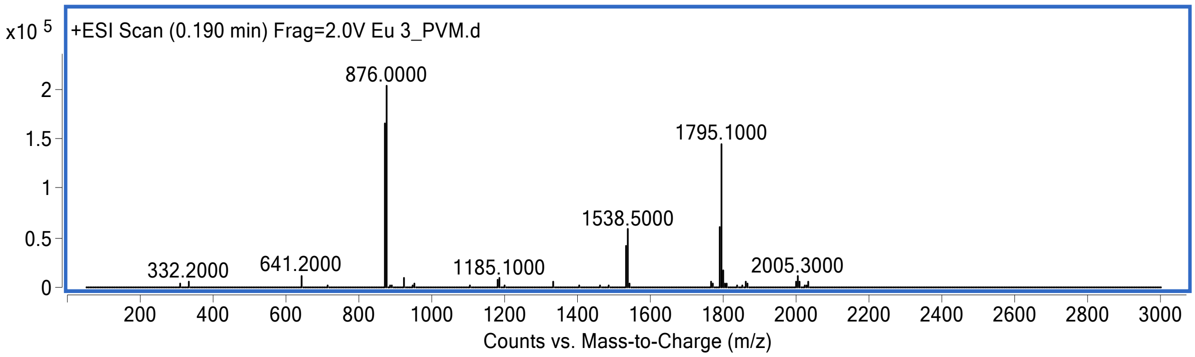

Figure S4: $^{+}$ESI-MS spectrum of **Eu-1** complex in DCM.

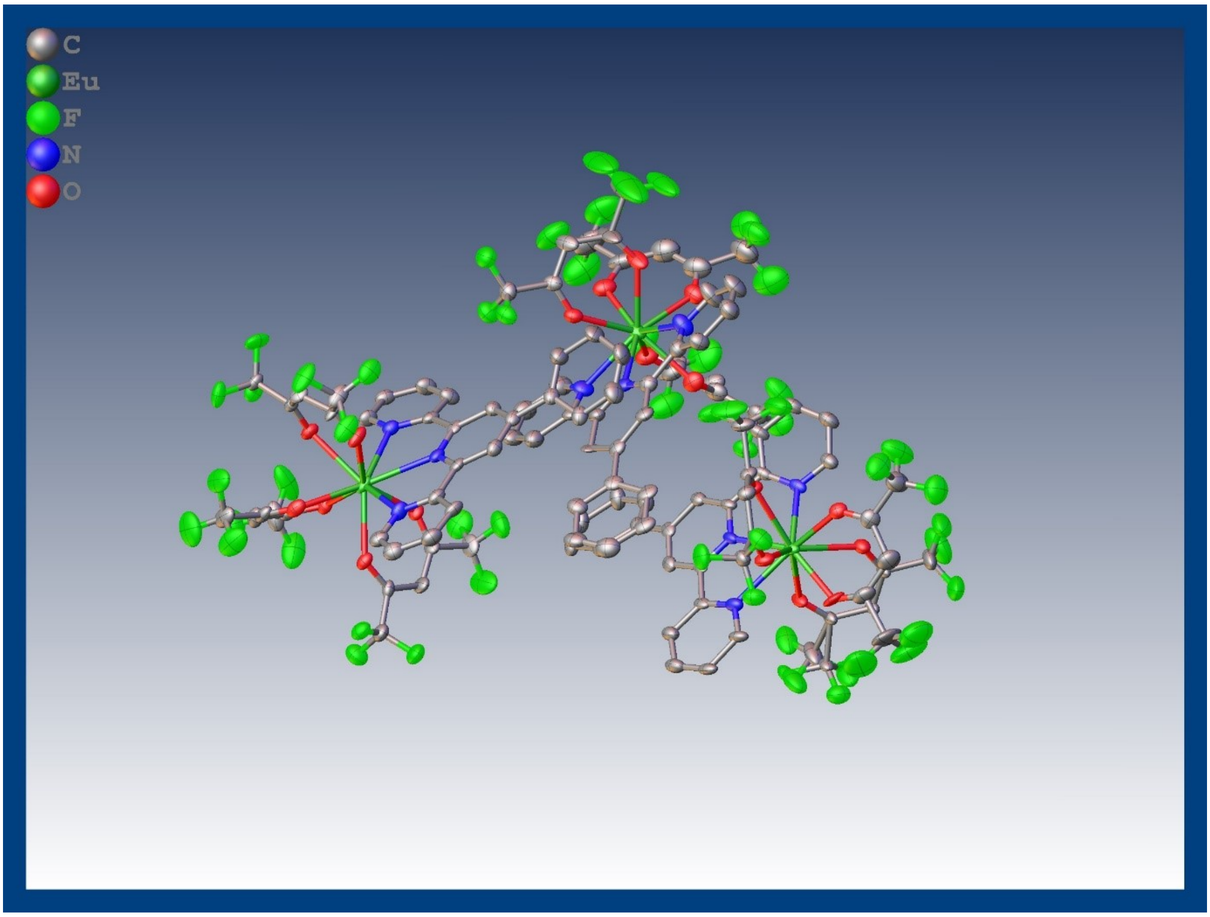

Figure S5: View of the asymmetric unit of **Eu-1.**

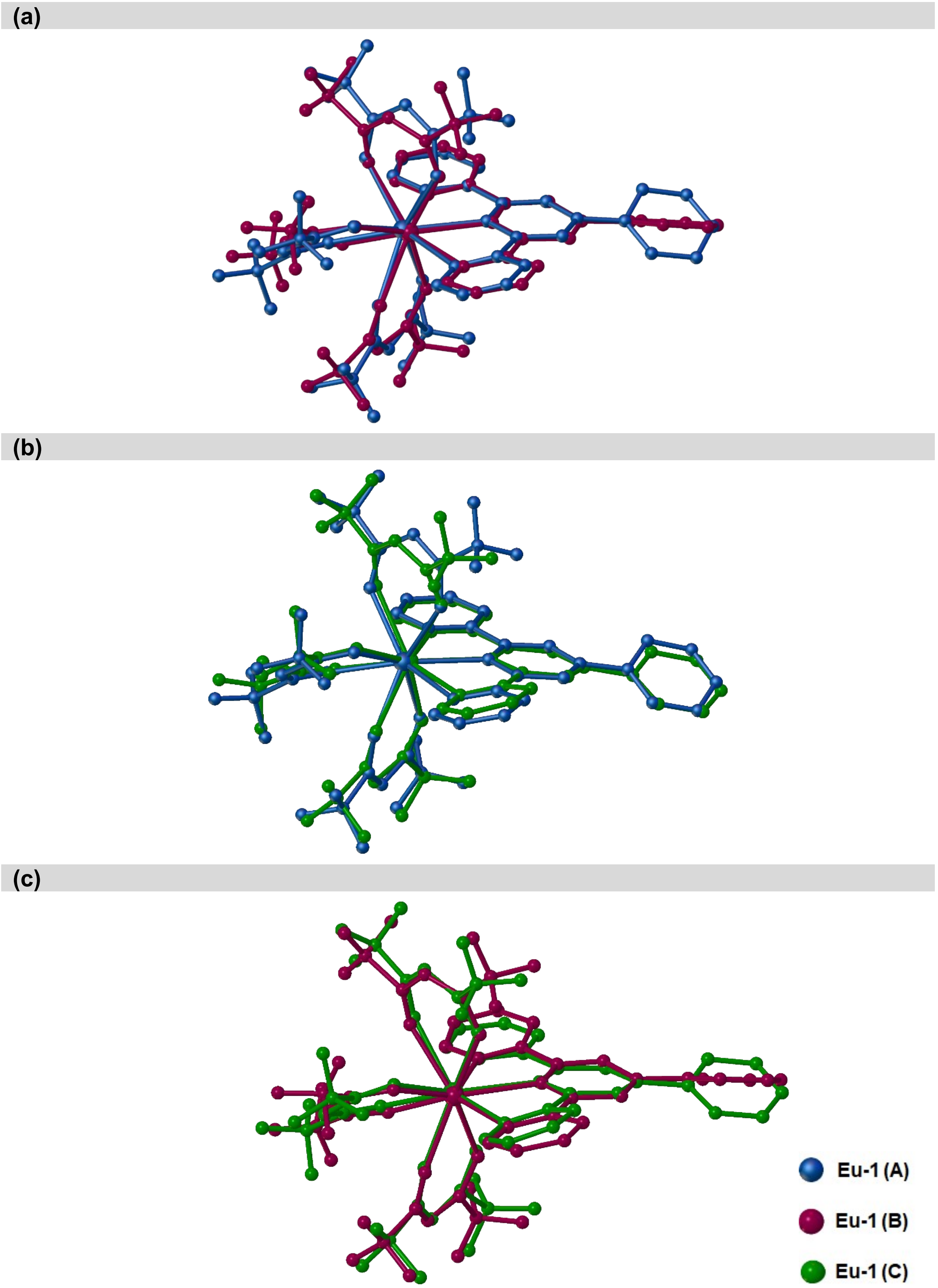

Figure S6: An overlap of (a) **Eu-1 (A)** and **Eu-1 (B)** (b) **Eu-1 (A)** and **Eu-1 (C)** and **(c) Eu-1 (B)** and **Eu-1 (C)**.

(a)

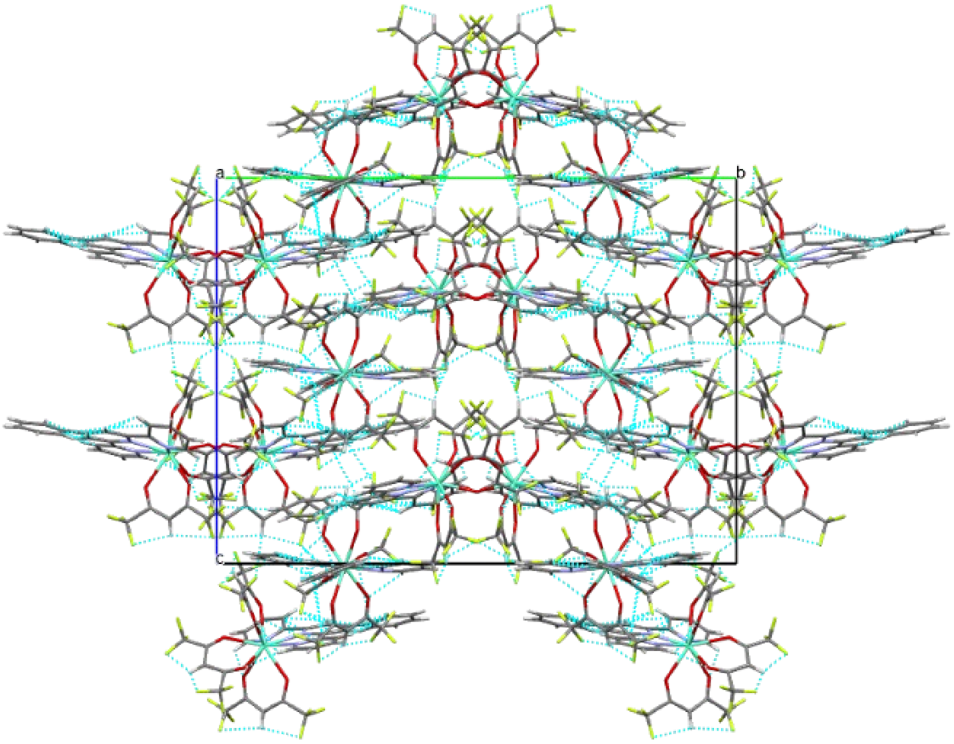

(b)

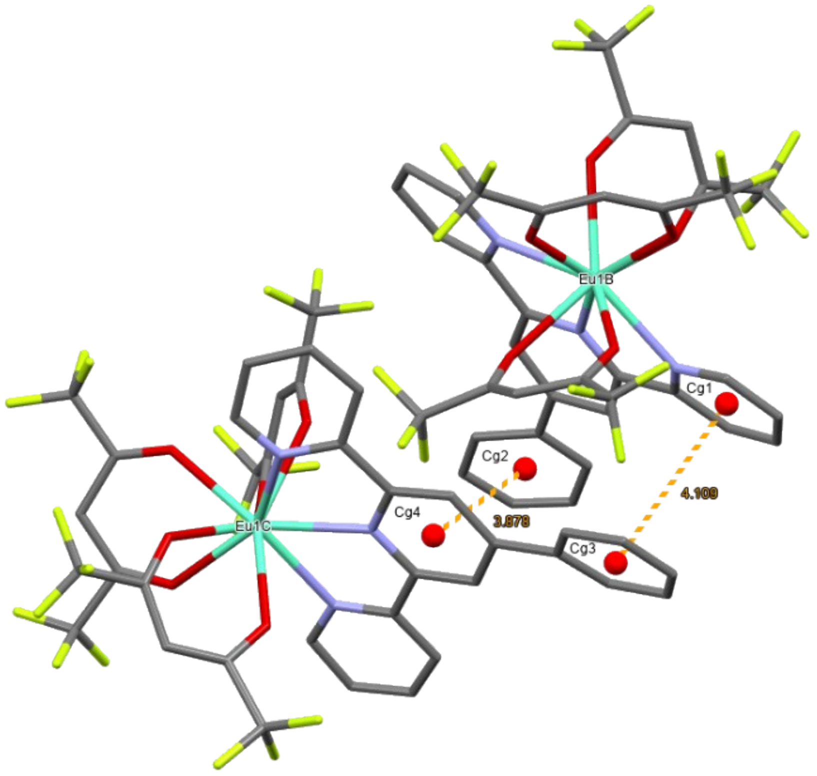

Figure S7: Crystal packing diagram of **Eu-1** illustrating **(a)** hydrogen bonding interactions **(b)** π-π stacking interactions.

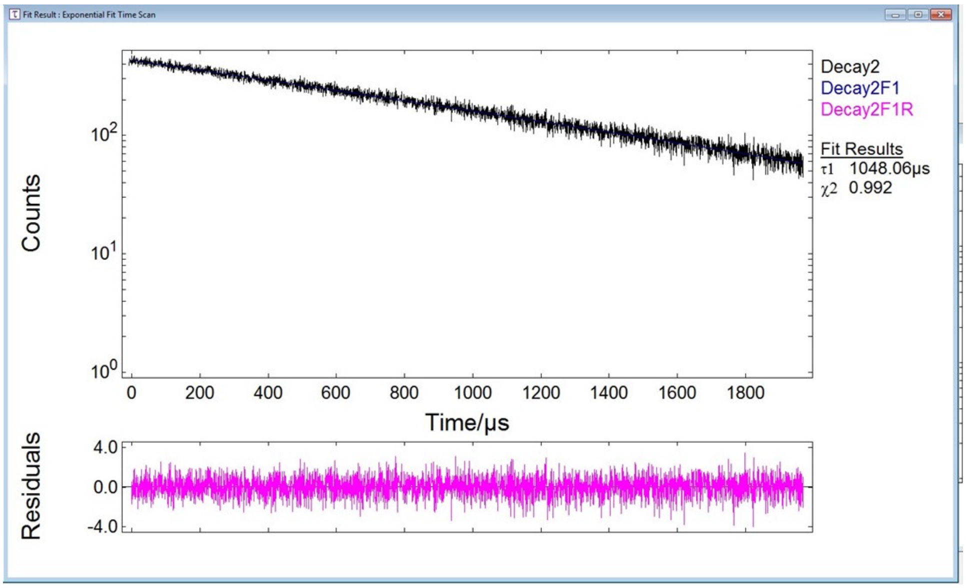

Figure S8: Room-temperature decay profile of **Eu-1** in DCM solution.

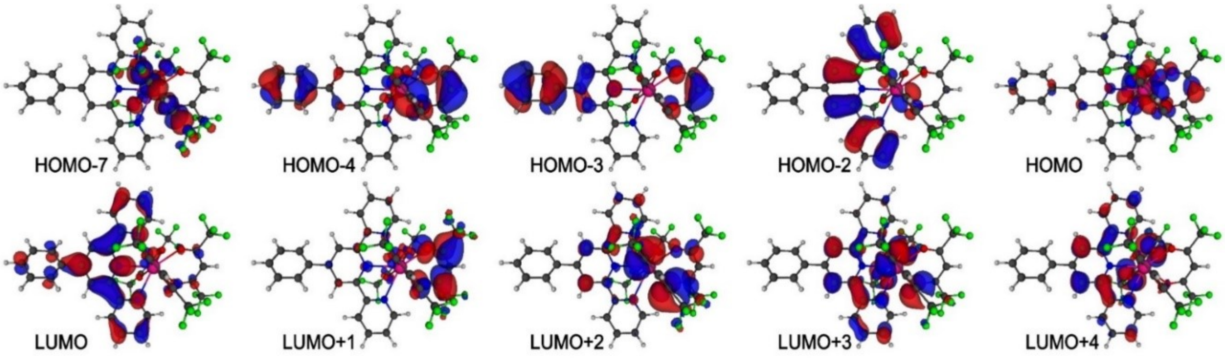

Figure S9: Selected molecular orbitals calculated with the PBE1PBE/TZVP/MWB52 TD-DFT method, accounting for the implicit effect of the DCM solvent.

References

1. O. V. Dolomanov, L. J. Bourhis, R. J. Gildea, J. A. K. Howard and H. Puschmann, *J. Appl. Crystallogr.*, 2009, **42**, 339-341.
2. G. Sheldrick, *Acta Crystallogr., Sect. A*, 2015, **71**, 3-8.
3. G. Sheldrick, *Acta Crystallographica Section A*, 2008, **64**, 112-122.
4. (a) M. S. Khan, R. Ilmi, W. Sun, J. D. L. Dutra, W. F. Oliveira, L. Zhou, W.-Y. Wong and P. R. Raithby, *J. Mater. Chem. C*, 2020, **8**, 5600-5612; (b) R. Ilmi, W. Sun, J. D. L. Dutra, N. K. Al-Rasbi, L. Zhou, P.-C. Qian, W.-Y. Wong, P. R. Raithby and M. S. Khan, *J. Mater. Chem. C*, 2020, **8**, 9816-9827; (c) R. Ilmi, D. Zhang, J. D. L. Dutra, N. Dege, L. Zhou, W.-Y. Wong, P. R. Raithby and M. S. Khan, *Org. Electron.*, 2021, **96**, 106216; (d) I. J. Al-Busaidi, R. Ilmi, J. D. L. Dutra, W. F. Oliveira, A. Haque, N. K. Al Rasbi, F. Marken, P. R. Raithby and M. S. Khan, *Dalton Trans.*, 2021, **50**, 1465-1477; (e) R. Ilmi,

J. Yin, J. D. L. Dutra, N. K. Al Rasbi, W. F. Oliveira, L. Zhou, W.-Y. Wong, P. R. Raithby and M. S. Khan, *Dalton T*, 2022, **51**, 14228-14242; (f) I. J. Al-Busaidi, R. Ilmi, D. Zhang, J. D. L. Dutra, W. F. Oliveira, N. K. Al Rasbi, L. Zhou, W.-Y. Wong, P. R. Raithby and M. S. Khan, *Dyes Pigm.*, 2022, **197**, 109879; (g) R. Ilmi, J. Wang, J. D. L. Dutra, L. Zhou, W.-Y. Wong, P. R. Raithby and M. S. Khan, *Chemistry – A European Journal*, 2023, **29**, e202300376; (h) R. Ilmi, X. Xia, J. D. L. Dutra, G. S. Santos, L. Zhou, W.-Y. Wong, P. R. Raithby and M. S. Khan, *ACS Applied Electronic Materials*, 2024, **6**, 2624-2638.

5. M. Dolg, H. Stoll, A. Savin and H. Preuss, *Theor. Chim. Acta*, 1989, **75**, 173-194.
6. F. Neese, *WIREs Comput. Mol. Sci.*, 2022, **12**, e1606.
7. C. Bannwarth, S. Ehlert and S. Grimme, *J Chem Theory Comput*, 2019, **15**, 1652-1671.
8. C. Bannwarth, E. Caldeweyher, S. Ehlert, A. Hansen, P. Pracht, J. Seibert, S. Spicher and S. Grimme, *WIREs Computational Molecular Science*, 2021, **11**, e1493.
9. M. A. Filho, J. D. L. Dutra, H. L. Cavalcanti, G. B. Rocha, A. M. Simas and R. O. Freire, *Journal of Chemical Theory and Computation*, 2014, **10**, 3031-3037.
10. J. J. P. Stewart, A. Klamt, W. Thiel, D. Danovich, G. B. Rocha, R. L. Gieseking, J. E. Moussa, H. A. Kurtz, P. Korambath, K. M. Merz and B. Wang, in *Zenodo*, Zenodo, Zenodo, 22.0.6 edn., 2022.
11. (a) O. L. Malta, *J. Lumin.*, 1997, **71**, 229-236; (b) F. R. G. E. Silva and O. L. Malta, *J. Alloys Compd.*, 1997, **250**, 427-430.
12. J. D. Dutra, T. D. Bispo and R. O. Freire, *J. Comput. Chem.*, 2014, **35**, 772-775.
13. A. N. Carneiro Neto, E. E. S. Teotonio, G. F. de Sá, H. F. Brito, J. Legendziewicz, L. D. Carlos, M. C. F. C. Felinto, P. Gawryszewska, R. T. Moura, R. L. Longo, W. M. Faustino and O. L. Malta, in *Handbook on the Physics and Chemistry of Rare Earths*, eds. J.-C. G. Bünzli and V. K. Pecharsky, Elsevier, 2019, vol. 56, pp. 55-162.
14. W. T. Carnall, H. Crosswhite and H. M. Crosswhite, *Energy level structure and transition probabilities in the spectra of the trivalent lanthanides in LaF_3*, United States, 1978.
15. E. Kasprzycka, A. N. Carneiro Neto, V. A. Trush, L. Jerzykiewicz, V. M. Amirkhanov, O. L. Malta, J. Legendziewicz and P. Gawryszewska, *J. Rare Earths*, 2020, **38**, 552-563.
16. J. D. L. Dutra, N. B. D. Lima, R. O. Freire and A. M. Simas, *Sci. Rep.*, 2015, **5**, 13695.
17. M. J. Weber, T. E. Varitimo and B. H. Matsinge, *Physical Review B*, 1973, **8**, 47-53.
18. A. J. Freeman and J. P. Desclaux, *J Magn Magn Mater*, 1979, **12**, 11-21.
19. O. L. Malta and F. R. G. E. Silva, *Spectrochim Acta A*, 1998, **54**, 1593-1599.
20. O. L. Malta, *J Non-Cryst Solids*, 2008, **354**, 4770-4776.
